# Subpopulation targeting of pyruvate dehydrogenase and GLUT1 decouples metabolic heterogeneity during collective cancer cell invasion

**DOI:** 10.1038/s41467-020-15219-7

**Published:** 2020-03-24

**Authors:** R. Commander, C. Wei, A. Sharma, J. K. Mouw, L. J. Burton, E. Summerbell, D. Mahboubi, R. J. Peterson, J. Konen, W. Zhou, Y. Du, H. Fu, M. Shanmugam, A. I. Marcus

**Affiliations:** 1 Graduate Program in Cancer Biology, Emory University, Atlanta, GA USA; 2 Winship Cancer Institute, Emory University, Atlanta, GA USA; 3 Department of Hematology and Medical Oncology, Emory University, Atlanta, GA USA; 4 Graduate Program in Molecular Systems Pharmacology, Emory University, Atlanta, GA USA; 5 Graduate Program in Biochemistry, Cell and Developmental Biology, Emory University, Atlanta, GA USA; 6 Department of Thoracic/Head & Neck Medical Oncology, MD Anderson Cancer Center, Houston, TX USA; 7 Department of Pharmacology and Chemical Biology, Emory University, Atlanta, GA USA; 8 Emory Chemical Biology Discovery Center, Emory University, Atlanta, GA USA

**Keywords:** Cancer metabolism, Metastasis, Collective cell migration

## Abstract

Phenotypic heterogeneity exists within collectively invading packs of tumor cells, suggesting that cellular subtypes cooperate to drive invasion and metastasis. Here, we take a chemical biology approach to probe cell:cell cooperation within the collective invasion pack. These data reveal metabolic heterogeneity within invasive chains, in which leader cells preferentially utilize mitochondrial respiration and trailing follower cells rely on elevated glucose uptake. We define a pyruvate dehydrogenase (PDH) dependency in leader cells that can be therapeutically exploited with the mitochondria-targeting compound alexidine dihydrochloride. In contrast, follower cells highly express glucose transporter 1 (GLUT1), which sustains an elevated level of glucose uptake required to maintain proliferation. Co-targeting of both leader and follower cells with PDH and GLUT1 inhibitors, respectively, inhibits cell growth and collective invasion. Taken together, our work reveals metabolic heterogeneity within the lung cancer collective invasion pack and provides rationale for co-targeting PDH and GLUT1 to inhibit collective invasion.

## Abstract (web summary)

The presence of phenotypic heterogeneity in collectively invading cells suggests cooperation amongst distinct subtypes of cells to promote invasion and metastasis. Here, the authors use chemical biology tools and report metabolic heterogeneity within the lung cancer collective invasion pack.

## Introduction

Next generation sequencing approaches have improved our understanding of genetic and epigenetic intra-tumor heterogeneity; however, the phenotypes created by this variable genetic landscape and heterogeneous microenvironment remain poorly understood^1–5^. This is in part due to approaches that rely on the bulk of the tumor population and consequently fail to provide information on how rare cellular sub-types function. This is especially true in the context of invasion and metastasis, where metastatic cells are likely a minor sub-type within the greater tumor cell population^6–8^. In most solid tumors, it is not single metastatic cells but rather collective packs of cells that are observed both histologically^9,10^ and as circulating tumor cell clusters^11^. These packs or clusters have greater metastatic efficiency compared to single cells^12^. Complementary mechanisms are likely at play in heterogeneous tumor cell packs, yet the molecular underpinnings driving the related phenotypic heterogeneity and cell:cell cooperation within these collective invasion packs has not been well studied.

To improve our understanding of phenotypic heterogeneity during collective invasion, we developed Spatiotemporal Cellular and Genomic Analysis (SaGA)^13^. This technique enables us to deconstruct cellular subpopulations based upon phenotype by observing live cells within a physiologically relevant environment. With SaGA, any observable cell(s) using a confocal microscope can be precisely selected and isolated based upon phenotypic criteria and subjected to a variety of downstream -omic analyses or further cultured for additional molecular studies. We previously used SaGA to probe the phenotypic heterogeneity within lung cancer collective invasion packs and isolated leader cells from the tips of invading chains, as well as the trailing follower cells. Our data revealed that phenotypically stable leader and follower subpopulations utilized an atypical angiogenic mimicry within collective invasion packs, which is similar but not identical to VEGF-driven angiogenesis among tip/stalk cells^13^. In the present study, we attempt to delineate the phenotypic heterogeneity within collective invasion packs by employing a chemical biology approach to probe cooperation among collectively invading cellular subtypes.

The data presented here reveal that lung cancer cell subpopulations within the collective invasion pack have distinct metabolic requirements. Leader cells rely on active pyruvate dehydrogenase (PDH) to sustain mitochondrial respiration whereas follower cells exhibit elevated glycolysis, glucose metabolism, glucose transporter 1 (GLUT1) expression, and reduced dependency on oxidative phosphorylation (OXPHOS). Here, we define this metabolic heterogeneity and propose that by exploiting the dependency of highly invasive cells on PDH and proliferative cells on GLUT1, we can co-target metabolic heterogeneity.

## Results

### Chemical biology screen identifies leader cell sensitivity to mitochondria-targeting agents

**Fig. 1:**
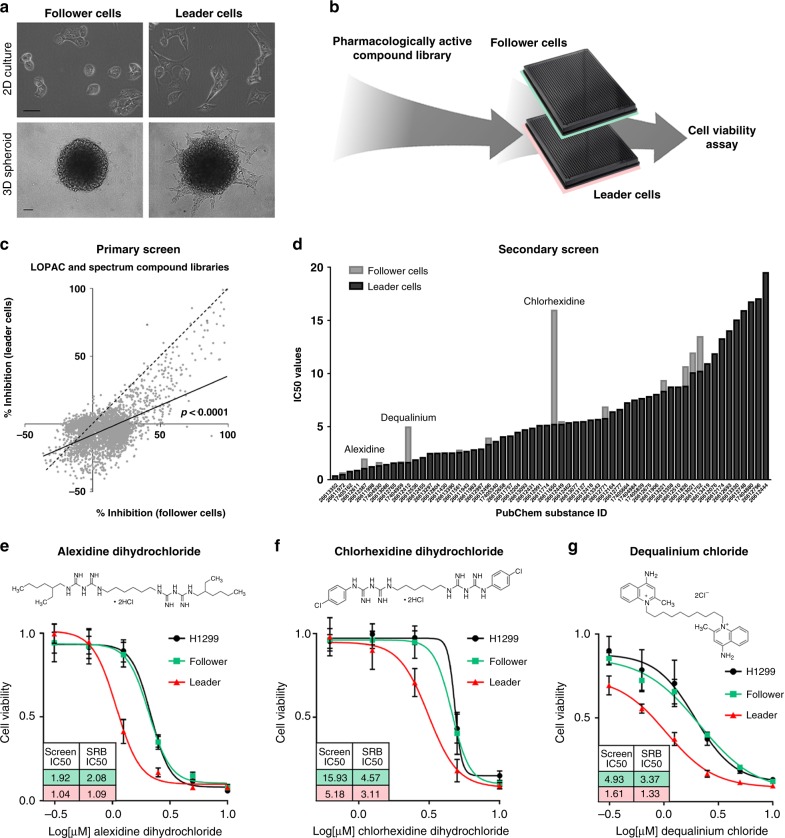
Chemical biology screen identifies leader cell sensitivity to mitochondria-targeting agents. **a** Representative brightfield images in 2D cell culture and in 3D cell spheroids embedded in Matrigel and allowed to invade for 24 h. Scale bar = 50 µm. Repeated three times independently with similar results. **b** For the chemical biology screen, each compound was tested for % inhibition of cell viability in 4 replicates at a final concentration of 14 μM in follower and leader cells. **c** Results of the primary compound screen are shown. Solid line = linear regression of % inhibition. *P* value represents the null hypothesis test of the regression shown and a regression with a slope of 1 being equal; *F* = 2576, DFn = 1, DFd = 6556, *p* < 0.0001. **d** Results of the secondary compound screen are shown, with follower cell IC_50_ values in light gray and leader cell IC_50_ values in dark gray for each PubChem Substance ID. **e**–**g** Alexidine, chlorhexidine, and dequalinium IC_50_ values were validated at final concentrations between 0.3 μM and 10 μM in an SRB assay. Follower cell IC_50_ values are shown in green and leader cell 72-h IC_50_ values are shown in pink. Error bars represent the mean ± SEM (*n* = 4 biologically independent samples). Chemical structures of alexidine dihydrochloride, chlorhexidine dihydrochloride, and dequalinium chloride are shown.

A specialized, highly invasive cellular subtype termed leader cells and a poorly invasive trailing subtype of follower cells were isolated from the collective invasion packs of the H1299 cell line utilizing SaGA as previously described^13^. Follower and leader cells maintain distinct morphologies in 2D and invasive phenotypes in 3D (Fig. 1a), whereby follower cells maintain this phenotype for a limited number of generations before reverting back to the parental phenotype and leader cells maintain their phenotype indefinitely. To probe the underlying biological differences of these subtypes, we conducted a chemical biology screen with two pharmacologically active compound libraries and evaluated the effects of each compound on cell viability (Fig. 1b). The primary screen of 3280 compounds at a single dose (14 μM) revealed that leader cells are globally resistant to most compounds compared to follower cells, including common chemotherapeutic agents (Figs. 1c, S1A), suggesting that leader cells cannot be targeted using standard therapeutics; however, leader cells were sensitive to antibiotics (Fig. S1B).

The primary screen compounds that demonstrated at least 50% inhibition of cell viability in either follower or leader cells were then tested in a secondary screen evaluating dose-dependent responses, where leader cells demonstrated greater sensitivity than follower cells to alexidine dihydrochloride, chlorhexidine dihydrochloride, and dequalinium chloride (Figs. 1d, S1C–E). These leader-specific agents share structural similarities in that they contain symmetrical biguanide (alexidine and chlorhexidine) or quinolinium (dequalinium) units tethered by a long alkyl chain and have each been reported to impair mitochondrial function^14–24^. Cell viability assays validated the sensitivity of leader cells to this class of compounds, and alexidine consistently had the lowest leader cell 72-h IC_50_ of ~1 μM (Fig. 1e–g). These findings show that leader cells are sensitive to mitochondria-targeting agents, particularly alexidine, and suggest that leader cells have a dependency on intact mitochondrial function.

### Alexidine dihydrochloride inhibits collective invasion and induces G1/G0 arrest

**Fig. 2:**
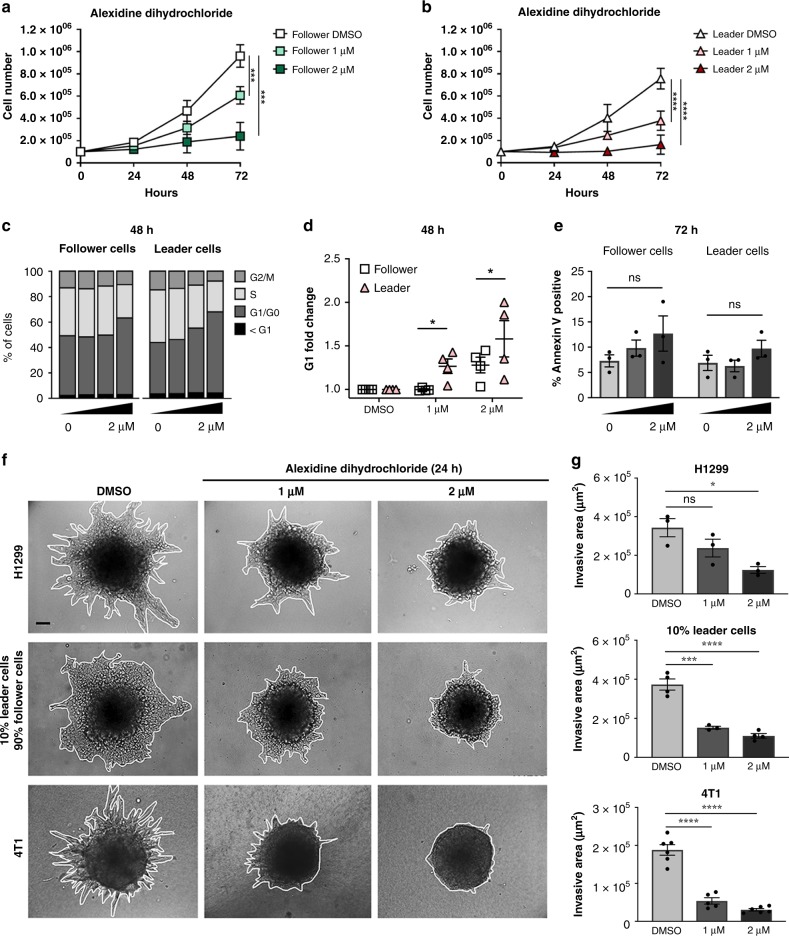
Alexidine dihydrochloride inhibits collective invasion and induces G1/G0 arrest. **a**, **b** Cell counts were determined for follower and leader cells treated with either DMSO or alexidine after 24, 48, and 72 h of treatment and trypan blue stained cells were excluded. Error bars represent the mean ± SEM (*n* = 3 biologically independent samples). An ordinary one-way ANOVA with a Tukey’s multiple comparisons test was used to determine significance of 72 h values compared to DMSO; Follower 1 µM *p* = 0.0006, Follower 2 µM *p* = 0.0001, Leader 1 µM *p* = 0.0010, Leader 2 µM *p* < 0.0001. **c** Cells were treated with alexidine for 48 hours then fixed and stained with DAPI and cell cycle analysis was performed (*n* = 4 biologically independent samples). **d** G1/G0 fold change was calculated from cell cycle analysis data (*n* = 4 biologically independent samples). A two-tailed unpaired Student’s t-test was used to analyze statistical significance between leader and follower cell groups; 1 µM *p* = 0.0185, 2 µM *p* = 0.0233. **e** Cells were treated with alexidine for 72 h then stained with AnnexinV and acquired by flow cytometry (*n* = 3 biologically independent samples). No significant change in AnnexinV positive % was observed. **f** Cell spheroids were embedded in either Matrigel or Type I collagen with either DMSO or alexidine and allowed to invade for 24 h. Brightfield representative images are shown. Solid lines designate outer perimeter. Scale bar = 50 µm. **g** Invasive area was quantified for each spheroid condition shown in (**f**) (*n* = 3 biologically independent samples). An ordinary one-way ANOVA with a Tukey’s multiple comparisons test was used to determine significance compared to DMSO; H1299 2 µM *p* = 0.0135, 10% Leader 1 µM *p* = 0.0001, 10% Leader 2 µM *p* < 0.0001, 4T1 1 µM *p* < 0.0001, 4T1 2 µM *p* < 0.0001.

**Fig. 3:**
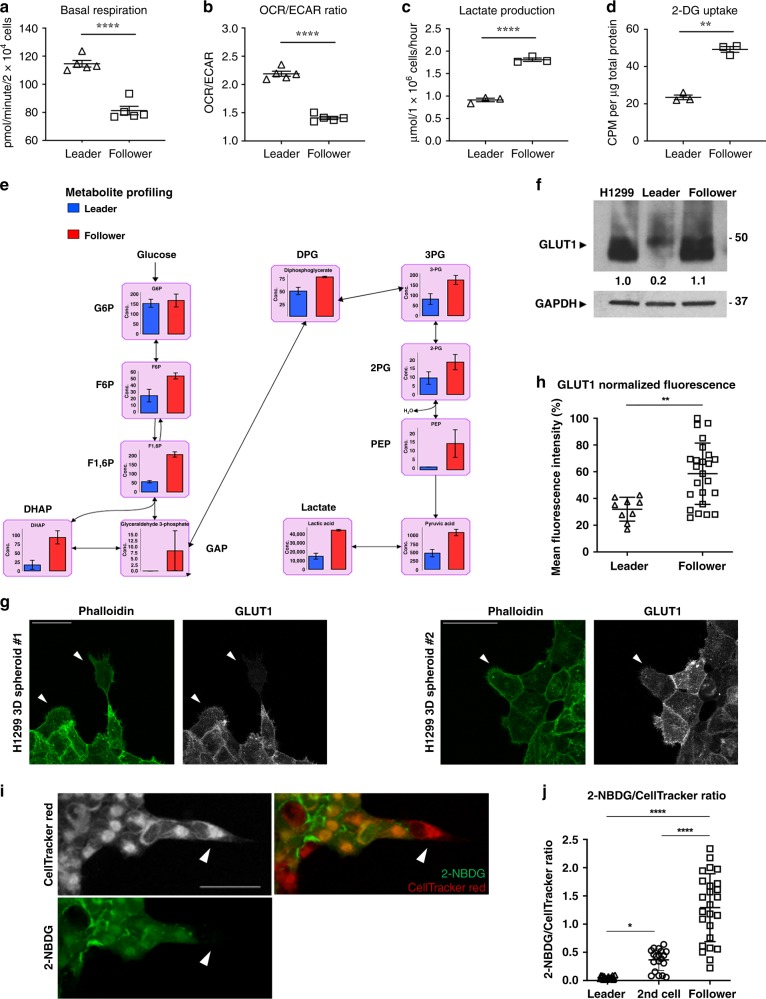
Follower cells exhibit elevated glucose uptake, glycolysis, and are less reliant on OXPHOS. **a** OCR, an indication of OXPHOS, and ECAR, an indication of glycolysis, were measured using Seahorse Bioscience XFe96 Analyzer. Basal respiration rate is calculated by subtracting OCR after the addition of antimycin and rotenone from the OCR before the addition of any inhibitors. Error bars represent the mean ± SEM (*n* = 5 biologically independent samples). A two-tailed unpaired Student’s *t*-test was used to analyze statistical significance between leader and follower cell groups; *p* < 0.0001. **b** Basal OCR/ECAR ratio was calculated and graphed. Error bars represent the mean ± SEM (*n* = 5 biologically independent samples). A two-tailed unpaired Student’s *t*-test was used to analyze statistical significance between leader and follower cell groups; *p* < 0.0001. **c** The lactate concentration in leader or follower cell culture media were evaluated. Error bars represent the mean ± SEM (*n* = 3 biologically independent samples). A two-tailed unpaired Student’s t-test was used to analyze statistical significance between leader and follower cell groups; *p* < 0.0001. **d** [^3^H]2-deoxyglucose uptake of leader cells and follower cells was measured at 37 °C for 6 min as described in the methods. Error bars represent the mean ± SEM (*n* = 3 biologically independent samples). A two-tailed unpaired Student’s *t*-test was used to analyze statistical significance between leader and follower cell groups; *p* = 0.0070. **e** Metabolic profiling of leader cells and follower cells was performed and the concentration of metabolites (pmol/10^6^ cells) in glycolysis pathway were graphed. Error bars represent the mean ± SEM (*n* = 3 biologically independent samples). **f** Cellular lysates were prepared and evaluated for GLUT1 protein expression with GAPDH as a loading control. Relative densitometry is normalized to H1299. Repeated three times independently with similar results. **g** Representative confocal images of invading chains from H1299 spheroids embedded in Matrigel and allowed to invade for 24 h, then fixed and stained with phalloidin and GLUT1. White arrowheads designate leader cells. Scale bars = 50 µm. **h** Results shown in (**g**) are quantified. Error bars represent the mean ± SEM (*n* = 9 cells examined over three independent experiments). A two-tailed unpaired Student’s *t*-test was used to analyze statistical significance between leader and follower cell groups; *p* = 0.0021. **i**) H1299 spheroids embedded in Martigel were allowed to invade for 24 h and then stained with CellTracker Red and 2-NBDG and fixed. Representative confocal images are shown. White arrowheads designate leader cells. Scale bar = 100 µm. **j** 2-NBDG and CellTracker Red fluorescence intensity were quantified and are shown here as a ratio of 2-NBDG normalized to CellTracker. Error bars represent the mean ± SEM (*n* = 19 cells examined over three independent experiments). An ordinary one-way ANOVA with a Tukey’s multiple comparisons test was used to determine significance; Leader and second cell *p* = 0.0424, Leader and follower *p* < 0.0001, second cell and follower *p* < 0.0001.

To better understand whether the observed decrease in cell number is due to cell cycle arrest or cell death, we performed Annexin V and DAPI staining. After 48 h of treatment, leader cells enter G1/G0 arrest at lower doses than follower cells (Figs. 2c, d and S2B, C), and after 72 h, there is no significant amount of cell death in either follower cells or leader cells (Figs. 3e and S2D, E). Additionally, there does not appear to be a significant amount of cell death with alexidine treatment in cells invading in 3D which express a cleaved caspase-3 apoptosis reporter (Fig. S2F). Altogether, these data suggest that G1/G0 arrest is responsible for the differential IC_50_ observed in leader and follower cells after alexidine treatment (Figs. 1e, S2A).

Leader and follower cell subpopulations cooperate to invade collectively in 3D^13^. Since alexidine was previously identified as an anti-metastatic agent in a 3D organotypic culture high throughput screen^25^, we tested the impact of alexidine on collective invasion in our system. Tumor cell spheroids were treated for 24 h, resulting in a dose-dependent inhibition of collective invasion across lung and breast cancer cell lines (Figs. 2f, g and S3A, B). However, alexidine treatment of 100% leader cell spheroids does not inhibit invasion in the same timeframe as a mixed spheroid population comprised of a lower leader cell percentage (Fig. S3C). Interestingly, G1/G0 arrest is likely not responsible for the anti-invasive activity of alexidine. We found that the decrease in invasiveness occurs prior to an effect on cell proliferation, which is not significant after 24 h of treatment (Fig. 2a, b), suggesting that the anti-invasive activity of alexidine is not due to the inhibition of proliferation. Additionally, alexidine inhibits invasion in transwell invasion assays (Fig. S4A–D), while in 2D it has a modest effect on motility and leader cell morphology (Fig. S4F–J). After 24 h of alexidine treatment, parental H1299 cells show a dose-dependent decrease in p-FAK^Y397^ and downstream p-MLC2^S19^ (Fig. S4E), both of which are part of the canonical cell motility machinery.

### Follower cells exhibit elevated glucose uptake, glycolysis, and are less reliant on OXPHOS

We next investigated the previously established role of alexidine in targeting cellular metabolism^17^ and the potential implications for collective invasion. We hypothesized that underlying differences in leader and follower cellular metabolism might contribute to their differential sensitivity to alexidine. As such, we assessed cellular metabolism in leader and follower cells. Our data show that follower cells exhibit lower basal respiration, take up more glucose, and produce more lactate than leader cells (Fig. 3a–d). Furthermore, we evaluated the basal oxygen consumption rate and extracellular acidification rate (OCR/ECAR) ratio as a measurement of relative OXPHOS vs glycolysis in both cells types. The higher OCR/ECAR ratio of leader cells (Fig. 3b) suggests a greater level of OXPHOS in leader cells and conversely, greater level of glycolysis in follower cells.

To further investigate glucose metabolism in both cell types, we preformed capillary electrophoresis-mass spectrometry (CE-MS)-targeted comparative metabolite profiling. Metabolite profiling was consistent with our previous observations, demonstrating increased glycolytic pathway intermediates in follower cells compared to leader cells (Fig. 3e). We detect an increase in key glycolytic intermediates in follower cells, including fructose-6-phosphate (F6P), fructose 1,6-bisphosphate (F1,6P), dihydroxyacetone phosphate (DHAP), diphosphoglycerate (DPG), 3-phosphoglyceric acid (3PG), 2-phosphoglyceric acid (2PG), phosphoenolpyruvate (PEP), pyruvate, and lactate. We also observed elevated ribose-5-phosphate (R5P) levels, a key pentose phosphate pathway (PPP) intermediate and precursor of ribonucleotides, and lower glucose-6-phosphate (G6P) to R5P ratio (Fig. S6A) suggesting upregulated PPP activity in follower cells. We next determined the reliance of follower and leader cells on OXPHOS by testing sensitivity to the Complex I inhibitors piericidin and metformin. In a 72-h cell proliferation assay, we detect elevated sensitivity of the leader cells to both these Complex I inhibitors suggesting increased dependency of leader cells on OXPHOS and follower cells on glycolysis (Fig. S6C). We then determined the effect of metformin on collective invasion and observed a significant decrease in chain-like invasion (Fig. S6D–F), suggesting that metformin also inhibits leader cell function.

To investigate the basis for elevated glucose uptake in follower cells we examined protein expression of the primary facilitative glucose transporter, GLUT1. We show higher GLUT1 expression in follower cells compared to leader cells, further supporting the observed higher glucose uptake and overall elevated glucose metabolism of follower cells (Fig. 3f). Importantly, we observe a marked decrease in GLUT1 expression in leader cells compared to follower cells during 3D collective invasion (Fig. 3g, h). We then measured uptake of 2-NBDG, a fluorescence-labeled 2-deoxy-glucose analog, and observed markedly higher 2-NBDG uptake in follower cells compared to leader cells as well as the cell directly behind the leader cell during invasion (Figs. 3i, j and S5). We also show higher expression of glucose-6-phosphate dehydrogenase (G6PD), which catalyzes the first rate-limiting step in the oxidative arm of the PPP, in follower cells (Fig. S6B). This observation is consistent with elevated R5P levels and an upregulated PPP in follower cells (Fig. S6A). Taken together, these results provide multiple lines of evidence for elevated GLUT1-driven glycolysis and PPP activity in follower cells compared to leader cells and the first line of evidence for metabolic heterogeneity within the lung cancer collective invasion pack.

### Alexidine dihydrochloride induces metabolic reprogramming of leader cells via S293 phosphorylation of PDH

**Fig. 4:**
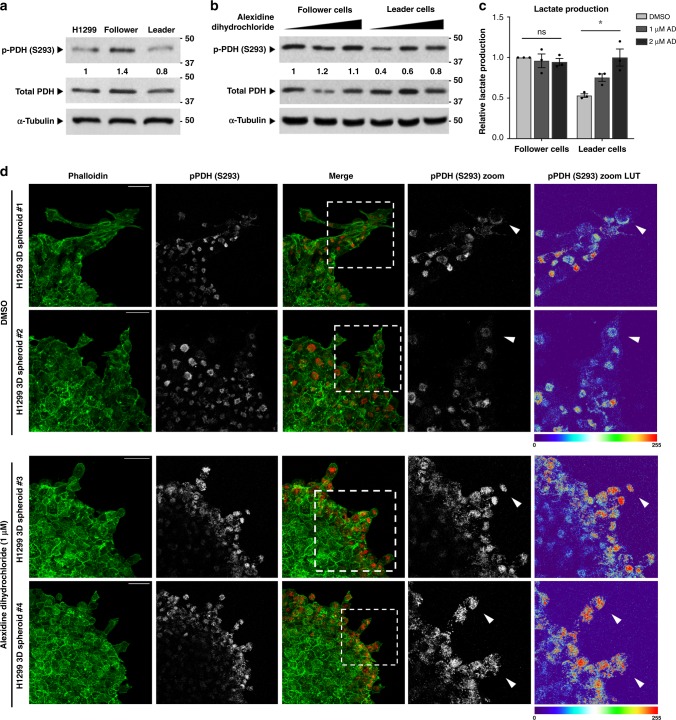
Alexidine dihydrochloride induces metabolic reprogramming of leader cells via S293 phosphorylation of PDH. **a** Cellular lysates were evaluated for p-PDH^S293^, total PDH, and α-Tubulin protein expression. Relative densitometry normalized is normalized to H1299. Repeated three times independently with similar results. **b** Cells were treated for 24 h and total protein lysates were evaluated for p-PDH^S293^, total PDH, and α-Tubulin protein expression. Relative densitometry normalized to DMSO treated follower cells is shown. Repeated 3 times independently with similar results. **c** The lactate concentration in leader or follower cell culture media were evaluated after 24 h of treatment with alexidine. Relative lactate production normalized to DMSO treated follower cells is shown. Error bars represent the mean ± SEM (*n* = 3 biologically independent samples). An ordinary one-way ANOVA with a Tukey’s multiple comparisons test was used to determine significance; *p* = 0.0110. **d** Confocal images of H1299 spheroids embedded in Matrigel and allowed to invade for 24 h, then fixed and stained with p-PDH^S293^ and phalloidin. Spheroids #1 and #2 were treated with DMSO and spheroids #3 and #4 were treated with alexidine for 24 h. Dashed boxes mark the zoom area. White arrowheads designate leader cells. Scale bars = 50 µm. Repeated three times independently with similar results.

Pyruvate dehydrogenase plays a gate-keeper role linking glycolysis to the citric acid cycle in the mitochondria. Since we show increased reliance of leader cells on OXPHOS, we next examined PDH’s activation state. We assessed S293 PDH phosphorylation, which inversely correlates with PDH activity and serves as a key node of metabolic regulation^26^. Consistent with previous results, follower cells showed higher inactivating p-PDH^S293^ supporting reduced citric acid cycle activity and OXPHOS, in contrast to the leader cells which had lower p-PDH^S293^ (Fig. 4a), providing more evidence for leader cell dependence on mitochondrial OXPHOS; however, we did not observe a clear pattern of mitochondrial activation in leader cells via tetramethylrhodamine methyl ester (TMRM) staining (Fig. S6G,H). This baseline difference in PDH phosphorylation between leader and follower cells may result from the differential mRNA expression of pyruvate dehydrogenase kinase isoform 4 (PDHK4), a key isoform responsible for phosphorylating and inactivating PDH^27,28^, which is more highly expressed in follower cells (Fig. S7A).

To determine whether alexidine can shift leader cells into glycolysis, we examined p-PDH^S293^ after 24 h of treatment. Leader cells show significant increase in p-PDH^S293^ while there is no significant impact on p-PDH^S293^ in follower cells (Figs. 4b, S7B). We also examined lactate production as a marker of glycolysis after 24 h of alexidine treatment, since baseline levels of lactate production were higher in follower cells compared to leader cells (Fig. 3d). We observe a dose-dependent increase in lactate production in leader cells after alexidine treatment while follower cells remain unaffected (Fig. 4c), further supporting the concept that alexidine shifts leader cells into glycolysis. Additionally, in highly metastatic 4T1 breast cancer cells, which demonstrate greater metabolic plasticity than the non-metastatic 67NR cells^29,30^, alexidine markedly increases p-PDH^S293^ (Fig. S7C, D). These findings indicate a shift toward glycolysis in leader cells as well as invasive 4T1 cells upon alexidine treatment that correlates with an induction of PDH phosphorylation.

To investigate whether PDH phosphorylation status is affected by alexidine during 3D collective invasion, we performed immunofluorescent staining of p-PDH^S293^ in fixed spheroids. These data show that in vehicle treated spheroids, p-PDH^S293^ expression follows a gradient, which is higher at the base of invading chains and lower in leader cells at the tips of those chains (Fig. 4d; spheroids #1 and #2). Whereas in the alexidine treated spheroids, the invading chains themselves are shorter, have a more rounded morphology, and higher p-PDH^S293^, especially in leader cells (4D; spheroids #3 and #4). These results establish a role for active PDH during 3D chain-like invasion, which can be inhibited using alexidine.

### PDH modulation drives invasive phenotype switching

**Fig. 5:**
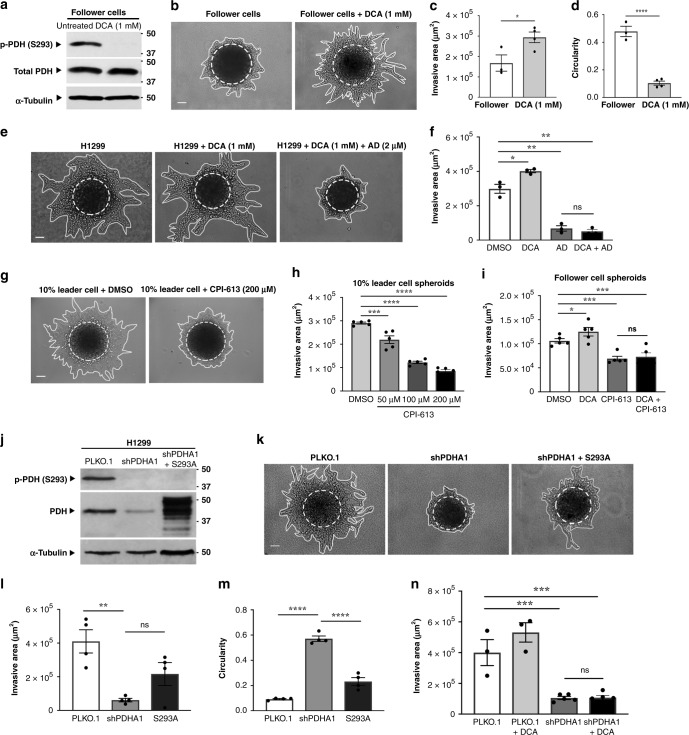
PDH modulation drives invasive phenotype switching. **a** Western blot of total protein lysates was probed for p-PDH^S293^, total PDH, and α-Tubulin after 24 h of DCA treatment compared to control. Repeated three times independently with similar results. **b** Follower cell spheroids were embedded in Matrigel with or without DCA and allowed to invade for 24 h. Brightfield representative images are shown. Solid lines designate outer perimeter and dashed lines designate inner spheroid core. Scale bar = 50 µm. **c** Invasive area for spheroids shown in (**b**) were quantified. Error bars represent the mean ± SEM (*n* = 3 biologically independent samples). A two-tailed unpaired Student’s *t*-test was used to analyze statistical significance; *p* = 0.0391. **d** Circularity was quantified from the invasive outer perimeter. Error bars represent the mean ± SEM (*n* = 3 biologically independent samples). A two-tailed unpaired Student’s *t*-test was used to analyze statistical significance; *p* < 0.0001. **e** H1299 cell spheroids were embedded in Matrigel with the treatments described and allowed to invade for 24 h. Scale bar = 50 µm. **f** Invasive area was quantified for the results shown in (**e**). Error bars represent the mean ± SEM (*n* = 3 biologically independent samples). An ordinary one-way ANOVA with a Tukey’s multiple comparisons test was used to determine significance compared to DMSO; *p* = 0.0196, *p* = 0.0019, *p* = 0.0014. **g** Spheroids formed from 90% follower cells and 10% leader cells were embedded in the presence of increasing doses of CPI-613 and were imaged after 24 h. Scale bar = 50 µm. **h** Quantification of invasive area from the experiment in (**g**). Error bars represent the mean ± SEM (*n* = 5 biologically independent samples). An ordinary one-way ANOVA with a Tukey’s multiple comparisons test was used to determine significance compared to DMSO; *p* = 0.0002, *p* < 0.0001, *p* < 0.0001. **i** Spheroid invasion assay on follower cells was performed in the presence of DCA (1 mM) or CPI-613 (200 μM) for 40 h. The invasive area is shown. Error bars represent the mean ± SEM (*n* = 5 biologically independent samples). An ordinary one-way ANOVA with a Tukey’s multiple comparisons test was used to determine significance compared to DMSO; *p* = 0.0164, *p* = 0.0004, *p* = 0.0009. **j** H1299 stable cell lines were generated with empty PLKO.1, shPDHA1, and PDH S293A as described in the methods. Western blot of total protein lysates was probed for p-PDH^S293^, total PDH, and α-Tubulin. Repeated three times independently with similar results. **k** Cell lines in (**j**) were embedded as spheroids and allowed to invade for 24 h. Scale bar = 50 µm. **l**, **m** Quantification of invasive area and circularity from the experiment shown in (**k**). Error bars represent the mean ± SEM (*n* = 4 biologically independent samples). An ordinary one-way ANOVA with a Tukey’s multiple comparisons test was used to determine significance; **l** *p* = 0.0033, **m** *p* < 0.0001, *p* < 0.0001. **n** H1299 shPDHA1 spheroids were treated with DCA for 24 h compared to control. Invasive area is show. Error bars represent the mean ± SEM (*n* = 3 biologically independent samples). An ordinary one-way ANOVA with a Tukey’s multiple comparisons test was used to determine significance compared to PLKO.1; shPDHA1 *p* = 0.0006, shPDHA1 with DCA *p* = 0.0007.

Because alexidine increases p-PDH^S293^ and inhibits collective invasion, we wanted to define the role of PDH in collective invasion. To do this, follower cells were treated with dichloroacetate (DCA), which indirectly activates PDH by inhibiting pyruvate dehydrogenase kinases (PDHKs)^31^. DCA treatment of follower cells decreased p-PDH^S293^ (Fig. 5a) and phenotypic analysis showed that DCA treatment induced a concomitant increase in invasion that is especially chain-like, similar to the leader cell phenotype (Fig. 5b–d). These findings suggest that the activation of PDH can promote collective invasion.

We postulated that alexidine could reverse the chain-like invasion phenotype via inhibition of PDH and treated cells with both alexidine and DCA, which led to a near total inhibition of all spheroid invasion compared to vehicle control or DCA alone (Fig. 5e, f). We also tested the clinically relevant PDH inhibitor CPI-613^32^. Similarly to alexidine, CPI-613 treatment led to a dose-dependent decrease in collective invasion (Fig. 5g, h). Furthermore, DCA plus CPI-613 treatment was unable to increase follower cell invasiveness (Fig. 5i), suggesting that the increased invasiveness observed with DCA alone is dependent upon PDH activation. To further support the necessity of active PDH for collective invasion, we depleted PDH via PDHA1 targeted shRNA as previously described^26^. In the absence of PDH, there is a significant decrease in collective invasion; however, introducing a constitutively active PDH harboring a S293A mutation partially restores the H1299 chain-like invasion phenotype (Fig. 5j–m), showing that collective invasion requires active PDH.

Lastly, to determine if DCA-induced invasion is indeed due to PDH activation, DCA was added to cells with PDH shRNA depletion. In this case, DCA was unable to promote invasion in the absence of PDH, supporting our findings that DCA-induced invasion is a result of PDH activation (Fig. 5n). These results demonstrate that PDH plays an essential role in chain-like collective invasion, which can be inhibited by using either alexidine or CPI-613.

### PDH is required to maintain pan-cytoplasmic mitochondrial distribution in invasive cells

**Fig. 6:**
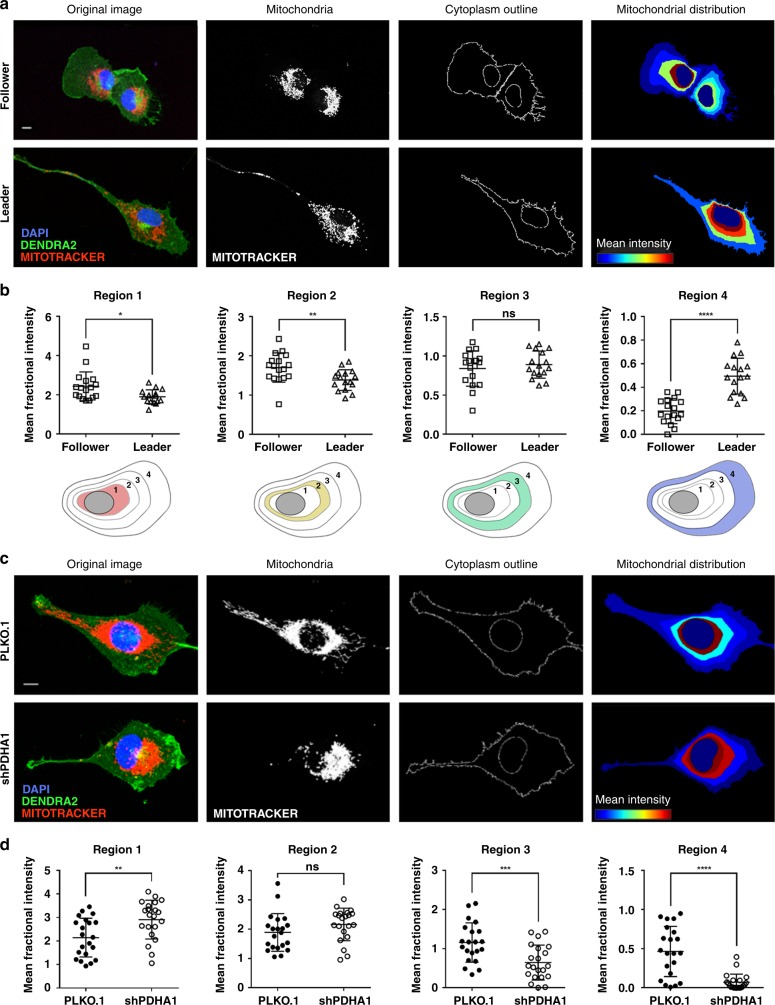
PDH is required to maintain pan-cytoplasmic mitochondrial distribution in invasive cells. **a** Live cells were stained with MitoTracker, fixed and mounted with ProLong with DAPI. Representative confocal images are shown. Scale bar = 100 µm. **b** Mitochondrial distribution analysis was performed in CellProfiler. Regions were defined from the edge of the nucleus to the outer edge of the cytoplasm and the mean fractional intensity of mitochondria was determined using the Measure Object Intensity Distribution module. The innermost perinuclear region (Region 1) to the outer cytoplasmic region (Region 4) are shown. Error bars represent the mean ± SEM (*n* = 20 cells over three independent experiments). A two-tailed unpaired Student’s *t*-test was used to analyze statistical significance between leader and follower cell groups; Region 1 *p* = 0.0202, Region 2 *p* = 0.0064, Region 4 *p* < 0.0001. **c** Live cells were stained with MitoTracker, fixed and mounted with ProLong with DAPI. Representative confocal images are shown. Scale bar = 10 µm. **d** Mitochondrial distribution analysis was performed in CellProfiler. Regions were defined from the edge of the nucleus to the outer edge of the cytoplasm and the mean fractional intensity of mitochondria was determined using the Measure Object Intensity Distribution module. The innermost perinuclear region (Region 1) to the outer cytoplasmic region (Region 4) are shown. Error bars represent the mean ± SEM (*n* = 20 cells over three independent experiments). A two-tailed unpaired Student’s *t*-test was used to analyze statistical significance between leader and follower cell groups; Region 1 *p* = 0.0039, Region 3 *p* = 0.0007, Region 4 *p* < 0.0001.

Enhanced mitochondrial respiration at the leading edge of invading cells has previously been described and facilitates focal ATP synthesis^33–36^, offering a potential explanation for the increased dependency on OXPHOS in highly invasive leader cells. Enhanced ATP synthesis and energy production can promote cell migration and invasion by supporting membrane protrusion and focal adhesion stability^36^. To determine whether leader and follower cells exhibit differential mitochondrial spatial organization, we analyzed mitochondrial distribution between the different subtypes (Fig. 6a, b). We found that leader cells have greater mitochondria distributed to the outer edges of the cytoplasm compared to follower cells, which have a predominantly perinuclear mitochondrial distribution. These data suggest that leader cells have increased mitochondrial trafficking throughout the cytoplasm, including the periphery (Fig. 6b; Region 4), while follower cells maintain their mitochondria in the perinuclear region.

Since we show that PDH is a key node for leader cell invasion and OXPHOS, we wanted to determine if the diffuse mitochondrial distribution observed in leader cells is PDH-dependent. When PDH was depleted in H1299 cells (Fig. 5j), we observed a shift in mitochondrial organization from pan-cytoplasmic to perinuclear distribution (Fig. 6c, d). These results show that PDH activity is associated with mitochondrial distribution to the cell periphery and indicate that pan-cytoplasmic mitochondrial distribution is a feature of highly invasive cells, whereas perinuclear mitochondrial distribution is a feature of poorly invasive cells.

### GLUT1 drives proliferation and suppresses collective invasion

**Fig. 7:**
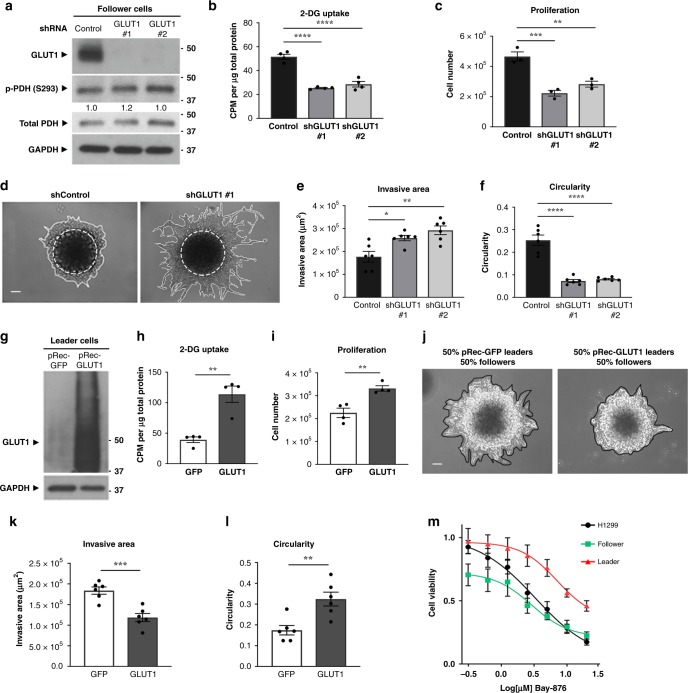
GLUT1 drives proliferation and suppresses collective invasion. **a** Follower cells were transduced with control shRNA or GLUT1-directed shRNAs and the efficiency of knockdown was determined by immunoblot analysis. GAPDH served as a loading control and blot was also probed for p-PDH^S293^ and total PDH. Relative densitometry is normalized to control. Repeated three times independently with similar results. **b** The uptake of [^3^H]2-deoxyglucose of cells from A was measured. Error bars represent the mean ± SEM (*n* = 4 biologically independent samples). An ordinary one-way ANOVA with a Tukey’s multiple comparisons test was used to determine significance compared to control; *p* < 0.0001, *p* < 0.0001. **c** Cells from A were evaluated for proliferation using trypan blue and an automated cell counter. Error bars represent the mean ± SEM (*n* = 3 biologically independent samples). An ordinary one-way ANOVA with a Tukey’s multiple comparisons test was used to determine significance compared to control; #1 *p* = 0.0006, #2 *p* = 0.0026. **d** Spheroid invasion assay was performed with cells from (**a**). **e**, **f** The invasive area and circularity are graphed from (**d**). Error bars represent the mean ± SEM (*n* = 6 biologically independent samples). An ordinary one-way ANOVA with a Tukey’s multiple comparisons test was used to determine significance compared to control; **e** #1 p = 0.0143, **e** #2 *p* = 0.0011, **f** #1 *p* < 0.0001, **f** #2 *p* < 0.0001. Scale bar = 50 µm. **g** Leader cells overexpressing GLUT1 were generated while leader cells overexpressing GFP served as a control. The expression of GLUT1 in these cells were evaluated by immunoblot analysis. Repeated three times independently with similar results. **h** The uptake of [^3^H]2-deoxyglucose of cells from (**g**) was measured. Error bars represent the mean ± SEM (*n* = 4 biologically independent samples). An ordinary one-way ANOVA with a Tukey’s multiple comparisons test was used to determine significance; *p* = 0.0019. **i** Cells from (**g**) were evaluated for proliferation using trypan blue and an automated cell counter. Error bars represent the mean ± SEM (*n* = 4 biologically independent samples). A two-tailed unpaired Student’s *t*-test was used to analyze statistical significance; *p* = 0.0040. **j** Spheroids formed from 50% follower cells and 50% leader cells from (**g**) were embedded and imaged. Scale bar = 50 µm. **k**, **l** The invasive area and circularity of the spheroids from in (j) are graphed in (**k**, **l**). Error bars represent the mean ± SEM (*n* = 6 biologically independent samples). A two-tailed unpaired Student’s *t*-test was used to analyze statistical significance; (**k**) *p* = 0.0006, **l** *p* = 0.0041. **m** Cell viability after 72 h of Bay-876 treatment at final concentrations between 0.3 and 20 μM was measured in an SRB assay. Error bars represent the mean ± SEM (*n* = 4 biologically independent samples).

Since follower cells showed increased 2-deoxyglucose (2-DG) uptake and GLUT1 expression compared to leader cells, we wanted to test if GLUT1 expression suppresses follower cell invasion. We depleted GLUT1 via GLUT1-targeted shRNAs (Fig. 7a) and observed a decrease in glucose uptake and proliferative capacity of follower cells (Fig. 7b, c). Importantly, we also observed a remarkable increase in overall invasion and chain-like collective invasion (decreased circularity; Fig. 7d–f). Interestingly, the effects of GLUT1 are not mediated by PDH activation (Fig. 7a), suggesting that GLUT1 reduction is sufficient to induce invasion in the follower context. We then tested if the reverse scenario is true by overexpressing GLUT1 in leader cells. In this case, GLUT1 overexpression increases glucose uptake and proliferation in leader cells and decreases collective invasion in follower and leader cell mixes (Fig. 7g–l). This is consistent with GLUT1 playing an anti-invasive but pro-proliferative role, where proliferation and collective invasion are inversely regulated by GLUT1.

Because follower cells more highly express GLUT1 (Fig. 3f), we hypothesized that follower cells are more sensitive to GLUT1 inhibition than leader cells. To test this, we treated cells with GLUT1 inhibitor Bay-876^37^ for 72 h. We found that follower cells and leader cells have differential sensitivity to Bay-876, where follower cells have a lower IC_50_ than leader cells in a viability assay (Fig. 7m).

### Alexidine dihydrochloride and Bay-876 co-target metabolic heterogeneity to inhibit collective invasion

**Fig. 8:**
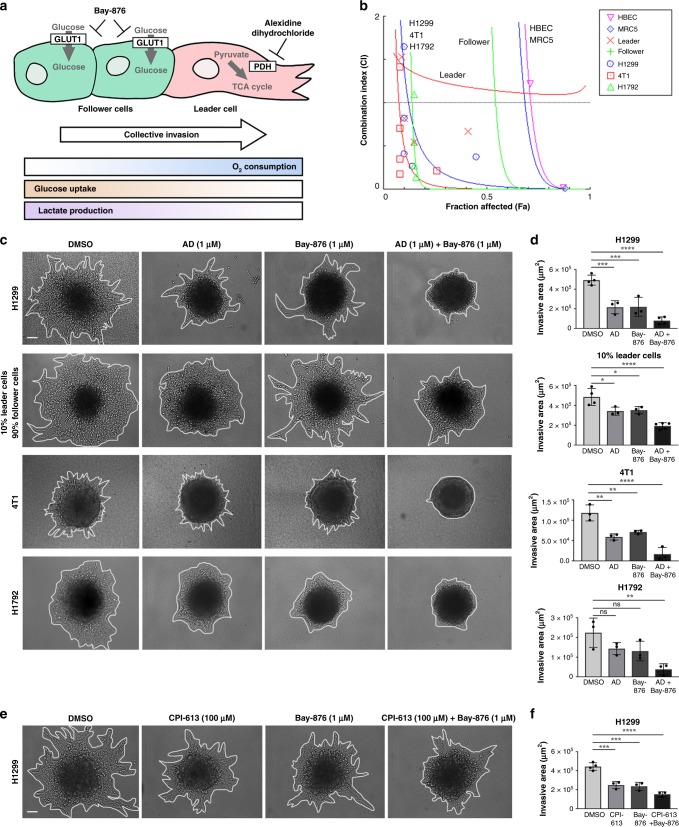
Alexidine dihydrochloride and Bay-876 co-target metabolic heterogeneity to inhibit collective invasion. **a** Overview of metabolically heterogeneous cellular subtypes in collective invasion and proposed co-targeting approach is shown. **b** Combination indexes were performed for parental lung and breast cancer cell lines (H1299, H1792, 4T1), lung subpopulation cell lines (follower and leader cells), and normal lung epithelial cells and lung fibroblasts (HBEC, MRC5) using a 72-h SRB assay for cell viability as described in the methods. (**C**) Cell spheroids were embedded in either Matrigel or Type I collagen with either DMSO, alexidine, Bay-876, or alexidine plus Bay-876 and allowed to invade for 24 h. Brightfield representative images are shown. Solid lines designate outer perimeter. Scale bar = 50 µm. **d** Invasive area was quantified for all conditions shown in (**c**). Error bars represent the mean ± SEM (*n* = 3 biologically independent samples). An ordinary one-way ANOVA with a Tukey’s multiple comparisons test was used to determine significance compared to DMSO; H1299 AD *p* = 0.0005, H1299 Bay *p* = 0.0006, H1299 AD + Bay *p* < 0.0001, 10% Leader AD *p* = 0.0140, 10% Leader Bay *p* = 0.0207, 10% Leader AD + Bay *p* < 0.0001, 4T1 AD *p* = 0.0017, 4T1 Bay *p* = 0.0068, 4T1 AD + Bay *p* < 0.0001, H1792 AD + Bay *p* = 0.0044. **e** H1299 spheroids were embedded in Matrigel with either DMSO, CPI-613, Bay-876, or CPI-613 plus Bay-876 and allowed to invade for 24 h. Scale bar = 50 µm. **f** Invasive area was quantified for all conditions shown in (**e**). Error bars represent the mean ± SEM (*n* = 3 biologically independent samples). An ordinary one-way ANOVA with a Tukey’s multiple comparisons test was used to determine significance compared to DMSO; H1299 CPI *p* = 0.0002, H1299 Bay *p* = 0.0001, H1299 CPI + Bay *p* < 0.0001.

Considering that alexidine targets active PDH in leader cells and Bay-876 targets GLUT1 in follower cells, we wanted to determine if the combination of alexidine and Bay-876 could co-target these cellular subtypes, respectively, during collective invasion (Fig. 8a). We used combination index analyses^38^ to determine synergy in cells treated with both compounds and found that alexidine and Bay-876 exhibit synergism in parental lung and breast cancer cell lines (Fig. 8b, Table S1). These data show that parental populations comprising multiple cellular subtypes are susceptible to drug combinations designed to co-target those subtypes. Importantly, synergism was observed at fewer dose combinations in normal lung epithelial cells or normal lung fibroblasts (Table S1), suggesting that this combination preferentially targets cancer cells. The combination of alexidine and Bay-876 is synergistic in leader cells and the parental cell lines, but not in follower cells (Table S1), suggesting that leader cells are sensitive to inhibition of GLUT1. We observe that alexidine primarily inhibits the viability of leader cells while Bay-876 primarily inhibits the viability of follower cells. We then examined the effects of Bay-876 plus alexidine on invasion and show that the combination inhibited collective invasion more than either compound alone across lung and breast cancer cell lines (Fig. 8c, d).

To test if the well-established PDH inhibitor CPI-613 would have a similar effect, Bay-876 and CPI-613 were combined to treat parental H1299 cell spheroids. Similarly to alexidine and Bay-876, the combination of Bay-876 and CPI-613 showed greater inhibition of collective invasion than either agent alone (Fig. 8e, f). CPI-613 (Devimistat) is being evaluated in various Phase I-II clinical studies as a single agent, as well as in combination with standard drug therapies, demonstrating that patients can tolerate a PDH inhibitor in combinatorial treatment strategies. Taken together, these results provide a rationale for co-targeting PDH and GLUT1 as a valid approach for inhibiting the collective invasion of heterogeneous cellular subtypes.

## Discussion

We performed the first chemical biology screen on specific cellular subtypes within the collective invasion pack and found that leader cells are globally resistant to the majority of compounds relative to follower cells (Fig. 1c). This is consistent with data showing that slow-cycling populations, like leader cells, are drug resistant^39^, and furthers reinforces the concept that the invasive and potentially metastatic cell subtypes are particularly difficult to target. These data also show that leader cells are sensitive to mitochondria-targeting agents, with alexidine being the top hit (Figs. 1 & S1). We show that alexidine promotes a glycolytic shift via PDH inactivation in leader cells and other highly invasive cells (Figs. 4 and S7), suggesting that leader cells are unable to invade upon impairment of OXPHOS and instead undergo G1/G0 arrest (Figs. 2 and S2).

While the effect of alexidine on metabolic reprogramming has been shown^17^, this is the first report showing that it induces p-PDH^S293^, thereby inactivating PDH (Fig. 4). Our data suggest that PDH activation in leader cells could be due to the marked decrease in PDHK4 expression in leader cells compared to follower cells (Fig. S6A). PDHK4 could be the PDHK isoform regulating PDH activity in this case^27,28^. This would be consistent with previous reports showing that PDHK4 expression mediates glycolysis and invasive capacity^40^ as well as lung tumorigenesis^41^. Decreased expression of PDHK4 in leader cells may be regulated by TGF-beta signaling, which promotes a mesenchymal transition via reduced expression of PDHK4^42^. Furthermore, a low level of p-PDH^S293^ at the invasive front of colorectal cancer lesions predicts tumor aggressiveness^43^, illustrating the prognostic value of PDH activity in patients. Our data suggest that PDH activity may be a unique vulnerability of invasive, leader-like cells that are dependent upon mitochondrial respiration and provides further support for targeting mitochondrial metabolism in cancer^44^.

Cancer cells exhibit altered glucose metabolism largely relying on glycolysis even in the presence of oxygen (Warburg effect)^45,46^; however, the metabolism of invasive and metastatic cancer cells can differ from the proliferating bulk of the tumor cell population^47–49^. While the downregulation of mitochondrial pathways is a common feature of metabolic rewiring across invasive cancer cells and is a poor prognostic indicator^50^, these studies focus on the bulk of the tumor cell population and would likely not detect rare, specialized subpopulations that are sensitive to mitochondrial inhibitors^51^. Maintaining intact mitochondrial function is considered essential for cancer cell invasion and metastasis to occur^52,53^, and enhanced OXPHOS, mitochondrial biogenesis, and oxygen consumption rates are observed in invasive breast cancer and pancreatic cancer^54–56^.

We show that leader cells demonstrate higher mitochondrial respiration, higher OCR/ECAR ratio, greater sensitivity to OXPHOS inhibition, reduced glucose uptake, reduced lactate production, and lower levels of p-PDH^S293^, all of which are indicative of elevated OXPHOS in leader cells (Figs. 3 and 4). We propose that PDH serves as a key metabolic switch since dysregulation of PDH through pharmacologic or genetic targeting has dramatic effects on invasion and causes invasive phenotype switching (Fig. 5). We show that PDH expression correlates with mitochondrial distribution to the cell periphery (Fig. 6), suggesting increased mitochondrial trafficking to the leading edge and offering an explanation for the reliance of highly invasive cells on active PDH and OXPHOS^33–36^. The change in mitochondrial distribution from the cell periphery to the perinuclear area reduces the ratio of ATP to ADP at the cell cortex, thereby impeding cell movement by impairing focal adhesion stability^36^. Leader cell dependence upon mitochondrial respiration may reflect a preference for more efficient ATP generation to sustain the energy required to invade, rather than the more inefficient aerobic glycolytic program required by highly proliferative cancer cells.

In contrast to leader cells, follower cells exhibit higher glucose uptake and express higher levels of GLUT1 (Figs. 3, S5). GLUT1, a ubiquitous glucose transporter in all tissues and the predominant glucose transporter in most cancer cells^57,58^, enables tumor cells to maintain glucose uptake and is associated with poor prognosis in various tumor types^59^ including lung cancer^60^. We show that GLUT1 suppression in follower cells induces a phenotypic switch to a more leader-like invasive pattern; conversely, forced GLUT1 expression in leader cells hinders invasion, showing that GLUT1 suppresses invasion in both leader and follower cells (Fig. 7). Higher GLUT1 expression also corresponds with higher glucose uptake and enhanced proliferation (Fig. 7). The decreased expression of GLUT1 in leader cells could be due to the regulation of multiple transcriptional regulators (Akt, HIF‐1, p53, c‐Myc) or the chromatin modifier HDAC2^61–65^. Glucose fuels glycolysis and is channeled into the PPP; enhanced glycolytic flux due to higher glucose uptake may be diverted to the PPP to support ribose biogenesis and promote proliferation without affecting the flux of citric acid cycle intermediates. The observation that GLUT1 suppression in follower cells does not activate PDH but increases invasiveness suggests that decreased entry of glycolytic intermediates into glycolysis and PPP is sufficient to shift follower cells from a proliferative program to an invasive one.

We show that invasive leader cells are less glycolytic compared to proliferative follower cells, establishing metabolic heterogeneity within the lung cancer collective invasion pack. One could speculate that maintaining metabolically heterogeneous cellular subtypes remains advantageous for tumor cell invasion and metastasis by providing the plasticity necessary to invade into a variable microenvironment and proliferate at distant sites. This diversification of metabolic dependencies could theoretically enable invading cells to better adapt to the selective pressures of the tumor microenvironment and maintain the ability to “go” and “grow” as a cooperative unit. While metabolic heterogeneity has been observed in solid tumors^66,67^ and metabolic cooperation has been described between cancer cells and the stroma^68–70^, this is the first report of metabolic cooperation within the lung cancer collective invasion pack.

Differential energetic dynamics during invasion have been described in breast cancer, highlighting the need to further investigate metabolic heterogeneity during collective invasion in a variety of contexts^71^. While Zhang et al. show that leader cells consume more glucose than followers, in our model leader cells consume significantly less glucose than followers (Figs. 3i, j and S5). We speculate that this difference could be due to molecular heterogeneity within and between cell types, the microenvironment, and various modes of migration, all of which could influence the metabolism of invading cells. An important consideration in addition to tumor type is the influence of the microenvironment on collective invasion. A hypoxic tumor microenvironment alters cellular metabolism and invasiveness through multiple mechanisms, including HIF-1 activation^72^, which in general promotes glycolysis, increased lactate production, and increased invasion^73^. Our observations that the leader and follower cell subpopulations have differential GLUT1 expression (downstream HIF-1 effector) and lactate production suggest that phenotypic heterogeneity within the invasion pack may in part be sustained by the microenvironment.

Our studies raise the interesting possibility of co-targeting glycolysis and mitochondrial metabolism within the heterogeneous tumor population by inhibiting both GLUT1 and PDH, which inhibits both cell viability and invasion (Fig. 8). While leader cells are less dependent upon glucose uptake than followers, we speculate that they still require some glucose to survive. Thus, adding Bay-876 to alexidine further inhibits leader cell viability (Fig. 8). Overall, we observe that alexidine preferentially inhibits the viability of leader cells while Bay-876 preferentially inhibits the viability of follower cells. In combination, these compounds inhibit collective invasion better than either single agent alone (Fig. 8). This suggests that distinct metabolic preferences are critical to regulating the proliferative vs invasive characteristics of the collective invasion pack, underscoring a potential need to target two distinct phenotypes to block the metabolic plasticity that can drive invasive cells.

## Methods

### Cell lines and transfections

H1299 and H1792 human NSCLC cells, 4T1 and 67NR murine breast cancer cells, and SUM159 human TNBC cells were cultured in Roswell Park Memorial Institute (RPMI-1640) media or Ham’s F12 media with hydrocortisone and insulin supplemented with 10% fetal bovine serum and 100 units/mL of penicillin and streptomycin and maintained at 37 °C and 5% CO_2_.

PDHA1 shRNA (Open Biosystems; 5′-CGAATGGAGTTGAAAGCAGAT-3′) was a gift from Jun Fan and Jing Chen (Emory University) and was stably transfected into H1299 cells using Lipofectamine 3000 (Invitrogen) followed by puromycin selection (2 mg/mL). The pFusionRed-PDHA1 expression vector (Evrogen) was modified by Oskar Laur (Emory Integrated Genomics Core) to ablate the PDHA1 shRNA target sequence and introduce a constitutively active S293A mutation. PDHA1 S293A was then stably transfected into H1299 shPDHA1 cells using Lipofectamine 3000 followed by G418 selection (350 µg/mL) and confirmed by RFP expression.

GLUT1 shRNAs (TRCN0000043583, TRCN0000043587) were purchased from Sigma-Aldrich. Leader-GFP and -GLUT1 expressing cells were generated^74^. Large-scale production of high-titer lentiviral vectors was carried out according to an established protocol^75^. For transduction, H1299 cells were plated in complete medium and centrifuged 8 µg/ml polybrene at 1500 g for 90 min at room temperature.

### Reagents and antibodies

Alexidine dihydrochloride, chlorhexidine dihydrochloride, dequalinium chloride, potassium dichloroacetate, and Bay-876 were purchased from MilliporeSigma and CPI-613 was purchased from Biovision. Total PDH antibody (9H9AF5) was obtained from ThermoFisher Scientific, p-PDH^S293^ (AP1062) and α-Tubulin (MAB1864) from MilliporeSigma, G6PD (ab91034) and GLUT1 (ab15309) from Abcam, and GAPDH (GTX41027) from GeneTex Inc. Total FAK antibody was from BD (610087), pFAK^Y397^ from Invitrogen (44-624 G), pMLC2^S19^ from Cell Signaling (3671), and GAPDH was from Cell Signaling (2118). Horseradish peroxidase (HRP)-conjugated secondary antibodies (Jackson ImmunoResearch) were used for western blotting.

### Chemical biology screen and viability assays

In the primary screen conducted by the Emory Chemical Biology Discovery Center (ECBDC), 3280 compounds were tested from the LOPAC^1280^ library from MilliporeSigma (1280 compounds) and the Spectrum Collection library from Microsource Discovery Systems, Inc. (2000 compounds). Follower and leader cells were seeded in 1536-well plates and incubated overnight. To account for the difference in proliferation rates, follower cells were plated 900 cells/well and leader cells were plated 1050 cells/well. For each compound, 0.1 µl of 1 mM library compounds were added to cells in four replicates at a final concentration of 14 μM and cells were incubated for 72 h. CellTiter-Blue (Promega) was added and the fluorescence intensity (FI) was measured using an Envision Multi-label plate reader after 4 h of incubation. The effect of each compound on the viability of cells was expressed as a percentage of DMSO treated control and calculated as the following: % of Control = (FI compound − FI blank)/(FI control − FI blank). % inhibition = 100 − % of Control.

For the secondary screen, the top 126 hit compounds out of the primary screen that exhibited at least 50% inhibition in either follower or leader cells were tested. Follower cells (1000 cells/well) and leader cells (1200 cells/well) were seeded in 384-well plates and incubated overnight. Each compound was tested in three replicates at final concentrations between 0.2 and 20 μM and cells were incubated for 72 h. Viability was measured as described above and dose curves for each compound were generated from which the IC_50_ values for each compound in both follower and leader cells were determined. To validate the IC_50_ values from the secondary screen, we performed SRB staining in 96-well plates after 72 h of treatment with each compound at final concentrations ranging from 0.3 to 10 μM.

### Cell cycle and cell death assays

For cell cycle analysis, cells were harvested and fixed in 95% ethanol at −20 °C before staining with DNA staining buffer (4 μg/mL DAPI, 0.25% Triton-X 100 in 1× PBS). DAPI staining was analyzed by flow cytometry on a BD FACSCanto-II cytometer using FACSDiva software. FlowJo software was used to exclude doublets and determine the distribution of cells with sub-G1, G1/G0, S, and G2/M peaks. For cell death analysis, cells suspended in culture media and adherent cells were harvested in EDTA-free trypsin and stained with AnnexinV Pacific blue then acquired by flow cytometry as described above. FlowJo software was used to measure AnnexinV positivity compared to unstained control.

### Spheroid formation and invasion assays

Spheroids were formed in ultra-low attachment 96-well round bottom plates (Corning) and embedded in either 2.0 mg/mL Matrigel (BD Biosciences) or 3.0 mg/mL rat tail Type I Collagen (BD Biosciences) in a 35 mm glass bottom dish (Cellvis) and allowed to invade for up to 72 h incubated at 37 °C. Images were taken using an Olympus IX51 microscope 10× (0.30 NA air) with an Infinity2 CCD camera. For drug treatments, compounds were added directly to the Matrigel or collagen during the embedding process, as well as to the growth media added on top of the matrix.

Invasive area was quantified by measuring both the total spheroid area around the outer perimeter and the inner spheroid core in ImageJ/Fiji and taking the difference between the two measures. Spheroid circularity was utilized as an indirect measure of the chain-like invasion pattern and was quantified in ImageJ/Fiji by measuring the spheroid outer invasive perimeter.

### Spheroid Immunofluorescence

Spheroids were washed once with 1× PBS containing calcium and magnesium warmed to 37 °C and then immediately fixed with PFA and glutaraldehyde (PBS containing calcium and magnesium with added 2% PFA and 0.001% glutaraldehyde; freshly prepared and warmed to 37 °C) for 20 min at room temperature. For immunofluorescence staining, permeabilization, three glycine rinses, blocking, and antibody staining were performed^76^. After primary and secondary antibody staining, spheroids were imaged with the Leica TCS SP8 inverted confocal microscope (X 40 oil HC PL APO, 1.30 NA) using 1.0 mm z-stack intervals, line scanning (405 nm DMOD Flexible, 488 nm argon, 561 nm DPSS, 633 nm Helium-Neon), and PMT detectors.

### Scratch wound and transwell invasion assays

For the in vitro scratch wound assay, 12-well plates were coated with 1 mg/mL fibronectin and cells were plated to create a confluent monolayer. A scratch was created with a p200 pipet tip and the respective treatments were added. Images were acquired at 0, 12, and 24 h using an Olympus IX51 microscope. For the transwell invasion assay, the invasive capacity was measured using the BD BioCoat Matrigel Invasion guidelines. Briefly, Boyden chamber inserts (ThermoFisher Scientific) were coated with 40 μl of Matrigel and allowed to solidify at 37 °C for 1 h. 5 × 10^4^ cells were seeded in triplicate in 0.1% FBS with appropriate treatments, while the lower chamber contained 10% FBS. Cells were allowed to invade through the porous membrane coated with Matrigel at 37 °C for 24 h. Inserts were fixed with 10% formalin and stained with 0.05% crystal violet. Cell counts were performed to quantify cell invasion.

### Metabolic assays

2-DG uptake: [^3^H]2-deoxyglucose uptake was determined^77^. Briefly, cells were first starved in glucose-free HEPES buffer for 30 min at 37 °C. [^3^H]2-deoxyglucose was added to a final concentration of 50 µM and the uptake was performed for 6 min at 37 °C then the reaction was quenched by washing with ice-cold PBS. Cells were lysed by incubating with PBS containing 1% Triton X-100 for 15 min and then centrifuged to remove cell debris. 500 µL of supernatant was added to 4 mL Scintisafe scintillation fluid (ThermoFisher Scientific #SX23-5) and ^3^H was counted. Remaining supernatant was used to determine protein concentration by the BCA assay (ThermoScientific Pierce BCA Protein Assay Kit #23225).

Live cell oxygen consumption and extracellular acidification rates: Oxygen consumption rate and extracellular acidification rates were measured using Seahorse bioscience extracellular flux (XFe96) analyzer^78,79^. The Agilent Seahorse XF Cell Mito Stress Test was performed according to the manufacturer’s user guide. In brief, leader or follower cells were seeded at a density of 20,000 cells per well of an XF96 cell culture microplate and incubated overnight to ensure attachment. Before the assay, cells were equilibrated in unbuffered Seahorse XF base medium supplemented with 10 mM glucose, 1 mM sodium pyruvate, and 2 mM glutamine in a non-CO_2_ incubator. Cellular metabolism was examined through sequential injections of oligomycin (2.5 µM), carbonyl cyanide 4-(trifluoromethoxy) phenylhydrazone (FCCP, 0.5 μM) and rotenone (2 μM)/antimycin A (2 μM). Basal respiration rate is calculated as baseline OCR subtracted by the OCR after the injection of antimycin and rotenone.

Lactate assay: Cells were grown in RPMI-1640 media supplemented with 11 mM glucose, 2 mM glutamine, 100 U/ml Penicillin and 100 µg/ml Streptomycin without FBS for 4 h. Lactate concentration in the media was measured using a Lactate Assay Kit (MilliporeSigma #MAK064) via the colorimetric detection method following the manufacturer’s instructions.

Metabolite profiling: Targeted quantitative analysis was performed on leader and follower cells in triplicates using capillary electrophoresis-mass spectrometry (CE-TOFMS, Agilent Technologies) to assess basal levels of metabolites by Human Metabolome Technologies, Inc. Samples were prepared following HMT’s Sample Preparation Protocol. Briefly, a flask of culture cell (4 × 10^6^ cells/sample) was used for the extraction of intracellular metabolites. The culture medium was aspirated from the dish and cells were washed twice by 5% mannitol solution (10 mL first and then 2 mL). The cells were then treated with 1000 µL of methanol and left at rest for 30 s in order to inactivate enzymes. Next, the cell extract was treated with 625 µL of Milli-Q water containing internal standards (H3304-1002, Human Metabolome Technologies, Inc., Tsuruoka, Japan) and left at rest for another 30 s. The extract was obtained and centrifuged at 2300 × *g* and 4 °C for 5 min and then 1000 µL of upper aqueous layer was centrifugally filtered through a Millipore 5-kDa cutoff filter at 9100 × *g* and 4 °C for 120 min to remove proteins. The filtrate was centrifugally concentrated for CE-TOFMS analysis.

2-NBDG uptake: For steady state 2-NBDG imaging in 3D, spheroids were embedded in a Matrigel and allowed to invade. After 24 h, 0.146 mM 2-NBDG (ThermoFisher Scientific #N13195) and CellTracker Red (ThermoFisher Scientific #C34552) were added to the media, and spheroids were allowed to invade an additional 24 h. Invaded spheroids were fixed with 2% PFA for 30 min at room temperature, and subsequently washed 4X for 30 min with PBS. For the glucose starve/stimulate 2-NBDG in 3D, spheroids were rinsed 1× with glucose-free RPMI. Spheroids were incubated with glucose-free RPMI. After 24 h of glucose-free RPMI culture, 2-NBDG was added to the media, and spheroids were returned to the incubator for 30 min. Invaded spheroids were fixed as described above. Image analysis of 2-NBDG uptake was done using ImageJ by measuring the thresholded mean intensity of individual cells within the invasion pack. A ratio was generated between the 2-NBDG and CellTracker Red mean intensities for each cell and then plotted.

### Western blot

Cellular protein expression was analyzed via western blotting^80^. For FAK signaling, EGF (20 ng/mL) was added for 15 min and cells were lysed with RIPA buffer with protease and phosphatase inhibitors. For GLUT1, cell lysates for protein evaluation were prepared with RIPA buffer supplemented with protease and phosphatase inhibitors and 1% PMSF and samples were not boiled before leading onto the polyacrylamide gel.

### Mitochondrial distribution analysis

Live cells were stained with MitoTracker (ThermoFisher Scientific #M22426) at a final concentration of 100 nM for 40 min then fixed and imaged using a Leica TCS SP8 inverted confocal microscope^80^. In order to determine the mitochondrial staining intensity distribution per cell, images were processed in CellProfiler 3.0.0 with a modified pipeline from Cataldo et al.^81^. utilizing the Measure Object Intensity Distribution function in four scaled bins from the outer edge of the nucleus (defined by DAPI staining) to the outer edge of the cytoplasm (defined by Dendra2 staining), which are described in the text as Regions 1–4 and illustrated in Fig. 6.

### Combination index analysis

Combination indexes were performed using SRB staining for cell viability as described above. Alexidine dihydrochloride or CPI-613 was combined with Bay-876 using the typical 2-drug combination at a constant ratio approach described by ComboSyn, Inc. Final concentrations for individual compounds ranged from 0.2 μM to 20 μM and the results were analyzed using freely available CompuSyn software^38^.

### Statistical analysis

A two-tailed unpaired Student’s t-test was used to analyze statistical significance between two conditions in an experiment. For experiments with three or more comparisons, an ordinary one-way ANOVA with a Tukey’s multiple comparisons test was used. Significance was assigned to *p* values <0.05; *p < 0.05, **p < 0.01, ***p < 0.001, ****p < 0.0001. Error bars represent the mean ± SEM.

### Reporting summary

Further information on research design is available in the Nature Research Reporting Summary linked to this article.

## Supplementary information

Supplementary Information

Reporting Summary

## Data Availability

All the data supporting the findings of this study are available within the article and its supplementary information files and from the corresponding author upon reasonable request. A reporting summary for this article is available as a Supplementary Information file.
