# Supplementary material for: Subpopulation targeting of pyruvate dehydrogenase and GLUT1 decouples metabolic heterogeneity during collective cancer cell invasion
Source: Nat Commun. 2020 Mar 24;11:1533. doi: 10.1038/s41467-020-15219-7 (PMC7093428; doi:10.1038/s41467-020-15219-7)

Supplementary Information

Subpopulation targeting of pyruvate dehydrogenase and GLUT1 to decouple metabolic heterogeneity during collective cancer cell invasion

R. Commander[1,†], C. Wei[2,†], A. Sharma[2], J.K. Mouw[2], L.J. Burton[2], E. Summerbell[1], D. Mahboubi[3], R.J. Peterson[4], J. Konen[5], W. Zhou[2,6], Y. Du[2,7,8], H. Fu[2,6,7,8], M. Shanmugam[2,6,*], A.I. Marcus[2,6,*]

[1]Graduate Program in Cancer Biology, Emory University, Atlanta, GA
[2]Winship Cancer Institute, Emory University, Atlanta, GA
[3]Graduate Program in Molecular Systems Pharmacology, Emory University, Atlanta, GA
[4]Graduate Program in Biochemistry, Cell and Developmental Biology, Emory University, Atlanta, GA
[5]Department of Thoracic/Head & Neck Medical Oncology, MD Anderson Cancer Center, Houston, TX
[6]Department of Hematology and Medical Oncology, Emory University, Atlanta, GA
[7]Department of Pharmacology and Chemical Biology, Emory University, Atlanta, GA
[8]Emory Chemical Biology Discovery Center, Emory University, Atlanta, GA
[†]These authors contributed equally to this work.
*Corresponding authors: Mala Shanmugam, (404) 727-3005, mala.shan@emory.edu;
Adam Marcus, (404) 778-4597, aimarcu@emory.edu

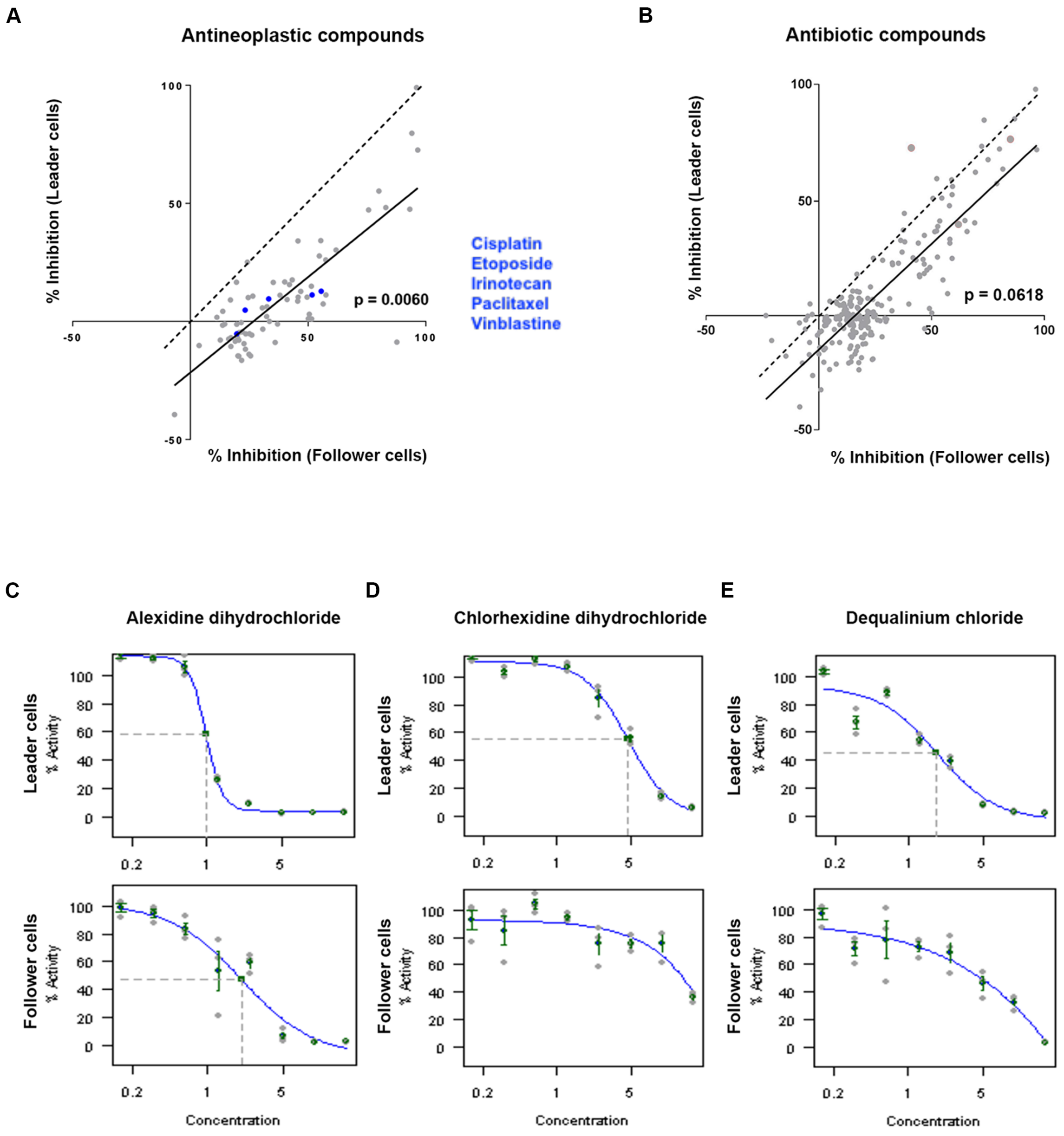

Supplementary Figure 1. Leader cells are sensitive to alexidine dihydrochloride, chlorhexidine dihydrochloride, and dequalinium chloride. (A) Primary screen results were filtered for antineoplastic compounds only, and chemotherapeutic agents are shown in blue. Solid line = linear regression of % inhibition, p value = difference between regression shown and regression with a slope of 1. (B) Primary screen results were filtered for antibiotic compounds only. Solid line = linear regression of % inhibition, p value = difference between regression shown and regression with a slope of 1, and the difference between regressions is not significant for antibiotics only. (C-E) For the secondary screen, 72-hour IC_{50} values were determined by testing each compound in 3 replicates at final concentrations between 0.2μM and 20μM and cell viability was measured by CellTiter-Blue. Dose curves for alexidine, chlorhexidine, and dequalinium are shown in follower and leader cells.

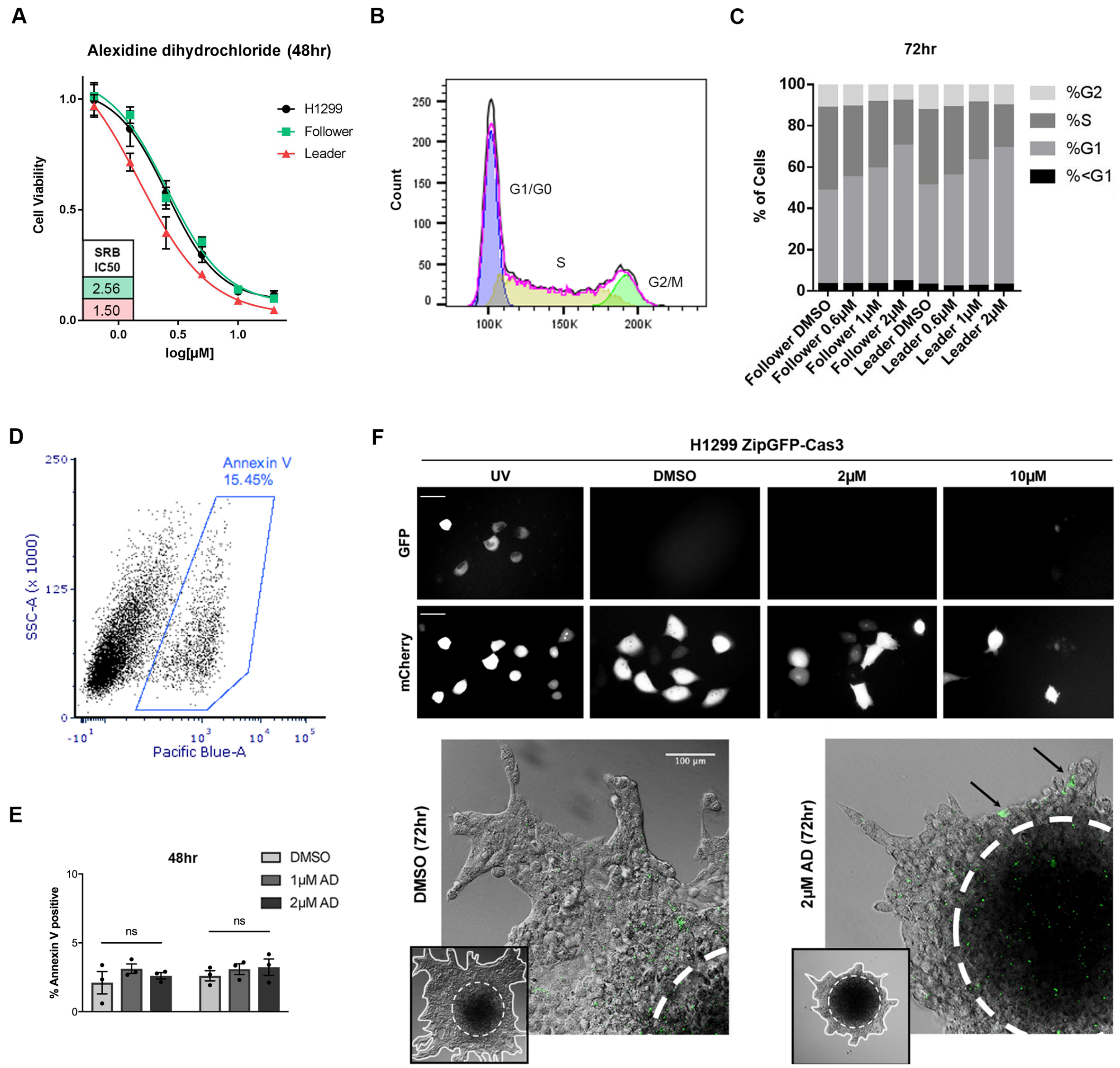

Supplementary Figure 2. Alexidine dihydrochloride decreases cell proliferation via G1/G0 arrest. (A) 48-hour IC_{50} values were determined at final concentrations between 0.3µM and 10µM in an SRB assay. Follower cell IC_{50} values are shown in green and leader cell 72-hour IC_{50} values are shown in pink. Error bars represent the mean ± SEM (n=4 biologically independent samples). (B) Representative plot of cell cycle analysis described in the methods. Untreated follower cells are shown. (C) Cells were treated with alexidine for 72 hours then fixed and stained with DAPI and cell cycle analysis was performed (n=3). (D) Representative plot of AnnexinV staining analysis and gating strategy described in the methods. (E) Cells were treated with alexidine for 48 hours then stained with AnnexinV and acquired by flow cytometry (n=3 biologically independent samples). No significant change in AnnexinV positive % was observed. (F) H1299 cells expressing ZipGFP-Cas3 apoptosis reporter construct were observed in 2D after exposure to UV for 5 minutes (positive control) or treatment with either DMSO or alexidine for 72 hours. Scale bar = 50µm. Representative images of 3D invasion in Matrigel with either DMSO or alexidine after 72 hours are also shown. Dashed lines designate inner spheroid core. Repeated 3 times independently with similar results.

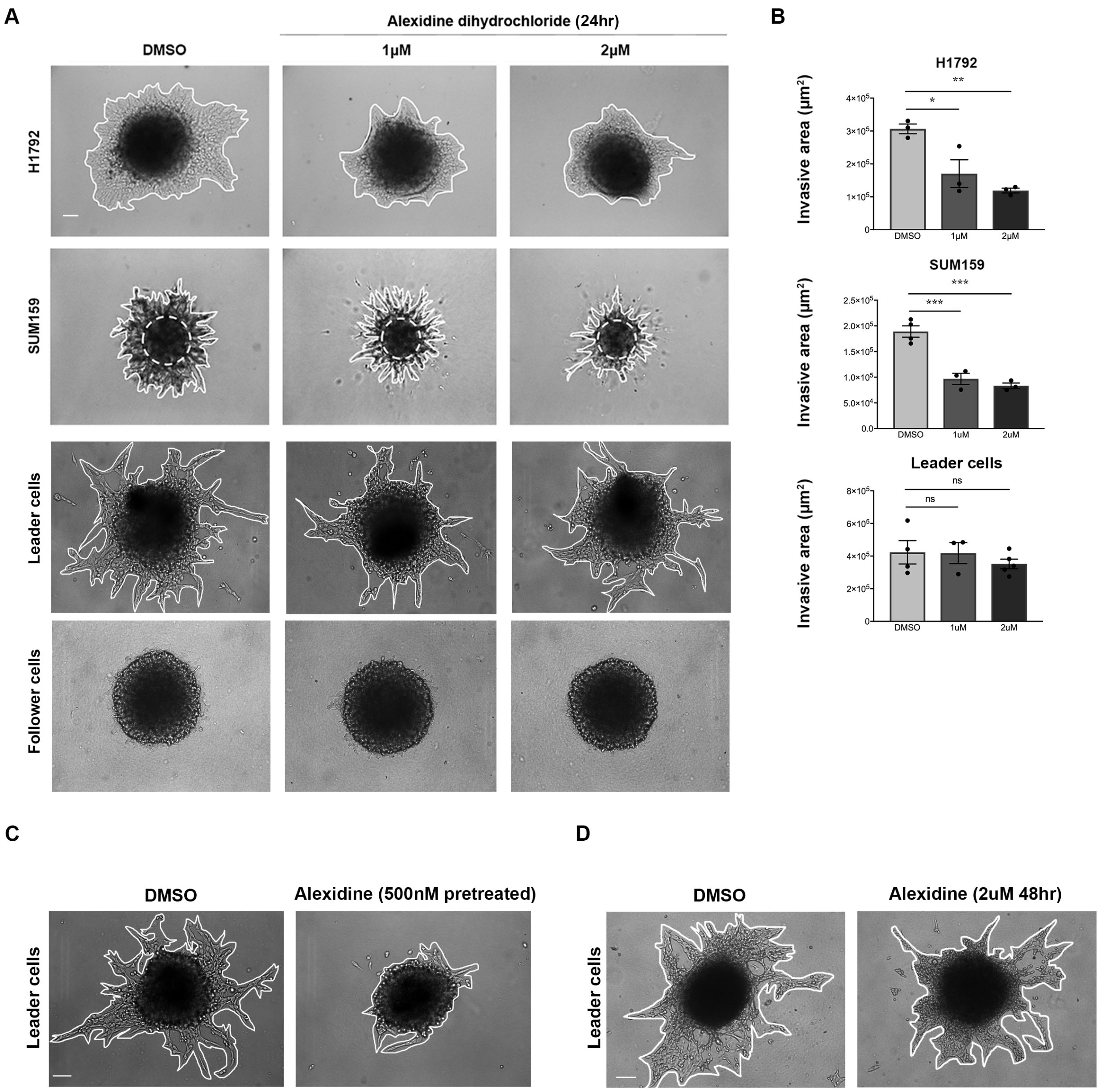

Supplementary Figure 3. Alexidine dihydrochloride decreases collective invasion across multiple cell lines. (A) Cell spheroids were embedded in either Matrigel or Type I collagen with either DMSO or alexidine and allowed to invade for 24 hours. Brightfield representative images are shown. Solid lines designate outer perimeter. Dashed lines designate inner spheroid core. Scale bar = 50μm. (B) Invasive area was quantified for each spheroid condition shown. Error bars represent the mean ± SEM (n≥3 biologically independent samples). An ordinary one-way ANOVA with a Tukey's multiple comparisons test was used to determine significance compared to DMSO; H1792 1μM $p=0.0182$, H1792 2μM $p=0.0041$, SUM159 1μM $p=0.0006$, SUM159 2μM $p=0.0003$. (C) Leader cells were pretreated with either DMSO or 500nM alexidine for 24 hours prior to being embedded in Matrigel, then allowed to invade for 24 hours. Scale bar = 50μm. Repeated 3 times independently with similar results. (D) Leader cells were embedded in Matrigel with either DMSO or 2μM alexidine then allowed to invade for 48 hours. Scale bar = 50μm. Repeated 3 times independently with similar results.

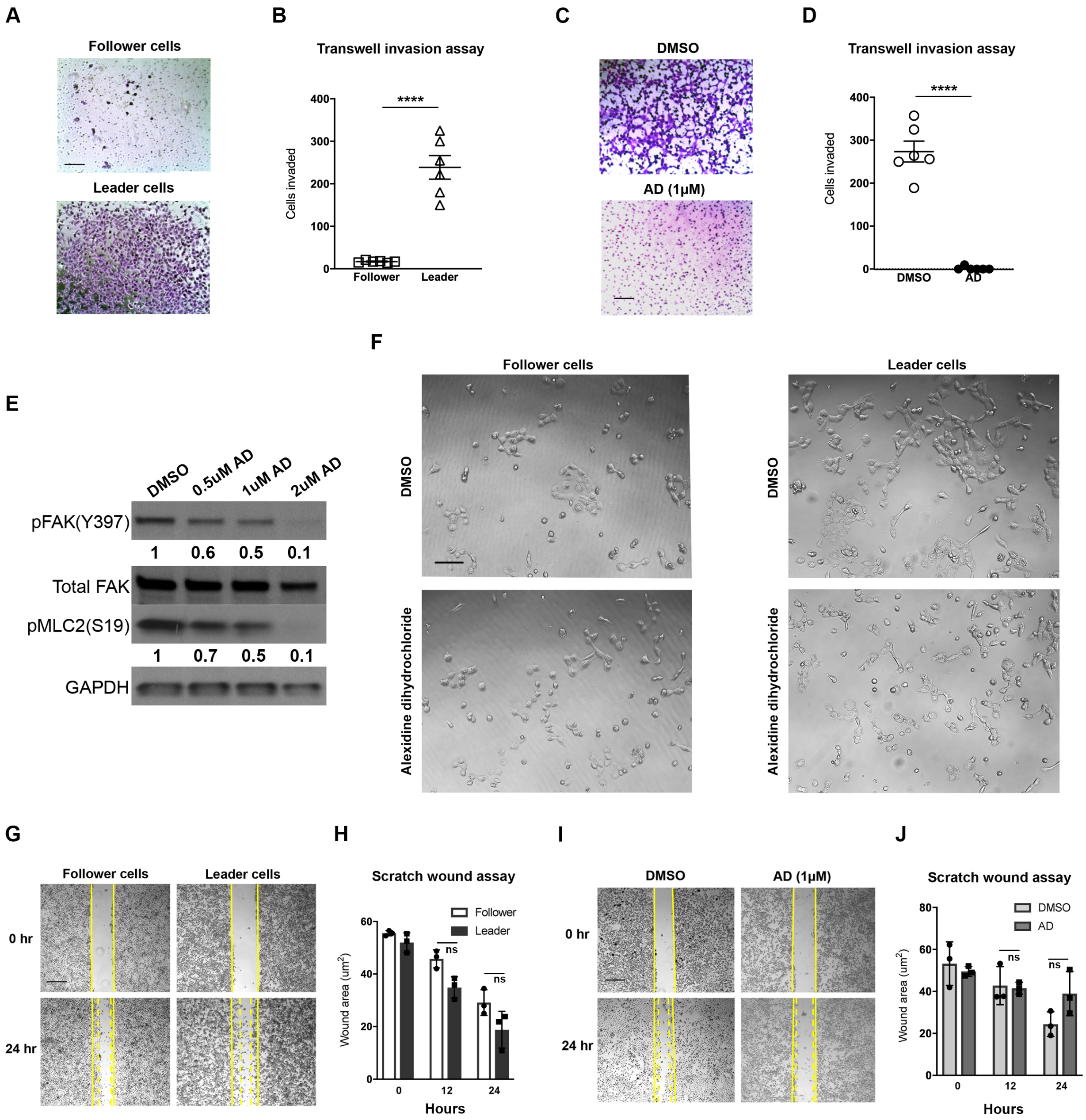

A
Follower cells
Leader cells
B
Transwell invasion assay
****
Cells invaded
400
300
200
100
0
Follower
Leader
C
DMSO
AD (1μM)
D
Transwell invasion assay
****
Cells invaded
DMSO
AD
E
DMSO
0.5uM AD
1uM AD
2uM AD
pFAK(Y397)
1 0.6 0.5 0.1
Total FAK
pMLC2(S19)
1 0.7 0.5 0.1
GAPDH
F
Follower cells
Leader cells
DMSO
Alexidine dihydrochloride
G
Follower cells
Leader cells
0 hr
24 hr
H
Scratch wound assay
Follower
Leader
ns
Wound area (um²)
Hours
I
DMSO
AD (1μM)
0 hr
24 hr
J
Scratch wound assay
DMSO
AD
ns
Wound area (um²)
Hours

Supplementary Figure 4. Alexidine dihydrochloride inhibits invasion in a transwell invasion assay. (A) Cells were allowed to invade through Boyden chambers coated with Matrigel at 37°C for 24 hours. Inserts were fixed and stained with 0.05% crystal violet for cells to be counted. Scale bar = 100μm. (B) Quantification of the number of cells invaded, with 4 fields of view averaged for each chamber counted (n=6 biologically independent samples). A two-tailed unpaired Student's t-test was used to analyze statistical significance; $p<0.0001$. (C) Cells treated with either DMSO or alexidine were allowed to invade through Boyden chambers coated with Matrigel at 37°C for 24 hours. Inserts were fixed and stained with 0.05% crystal violet for cells to be counted. (D) Quantification of the number of cells invaded, with 4 fields of view averaged for each chamber counted (n=6 biologically independent samples). A two-tailed unpaired Student's t-test was used to analyze statistical significance; $p<0.0001$. (E) H1299 cells were treated with either DMSO or alexidine for 24 hours and cell lysates were evaluated for $pFAK^{Y397}$, total FAK, $pMLC2^{S19}$, and GAPDH protein expression. Relative densitometry is normalized to control. Repeated 3 times independently with similar results. (F) Brightfield images of follower cells and leader cells in 2D culture with DMSO or alexidine after 24 hours of treatment. Repeated 3 times independently with similar results. Scale bar = 100μm. (G) Cells were plated to create a confluent monolayer on fibronectin coated plates. A scratch was created with a p200 pipet tip and images were acquired at 0,12, and 24 hours. Solid lines mark 0hr wound margins and dashed lines mark 24hr wound margins. Scale bar = 500μm. (H) Wound area quantification is shown (n=3 biologically independent samples). (I) Cells were plated to create a confluent monolayer on fibronectin coated plates and treated with DMSO or alexidine. A scratch was created with a p200 pipet tip and images were acquired at 0,12, and 24 hours. Solid lines mark 0hr wound margins and dashed lines mark 24hr wound margins. (J) Wound area quantification (n=3 biologically independent samples).

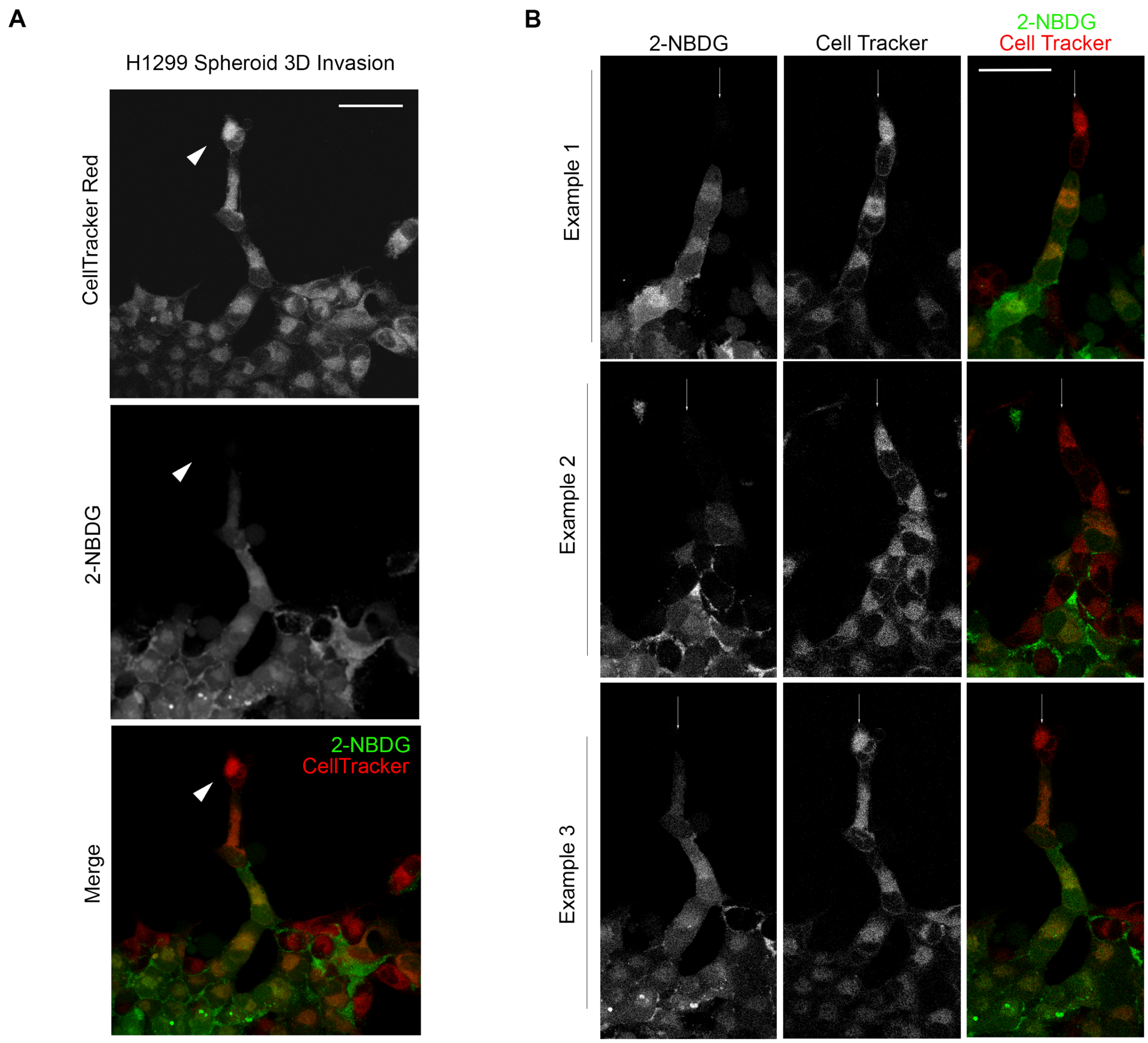

Supplementary Figure 5. Follower cells show increased 2-NBDG uptake during 3D collective invasion. (A) H1299 spheroids embedded in Martigel were allowed to invade for 24 hours and then stained with CellTracker Red and 2-NBDG and fixed. Representative confocal images are shown. White arrowheads designate leader cells. Scale bar = 50µm. Repeated 3 times independently with similar results. (B) Representative images of individual chains stained with 2-NBDG and CellTracker Red that were quantified in Supplementary Figure 3J. Scale bar = 50µm.

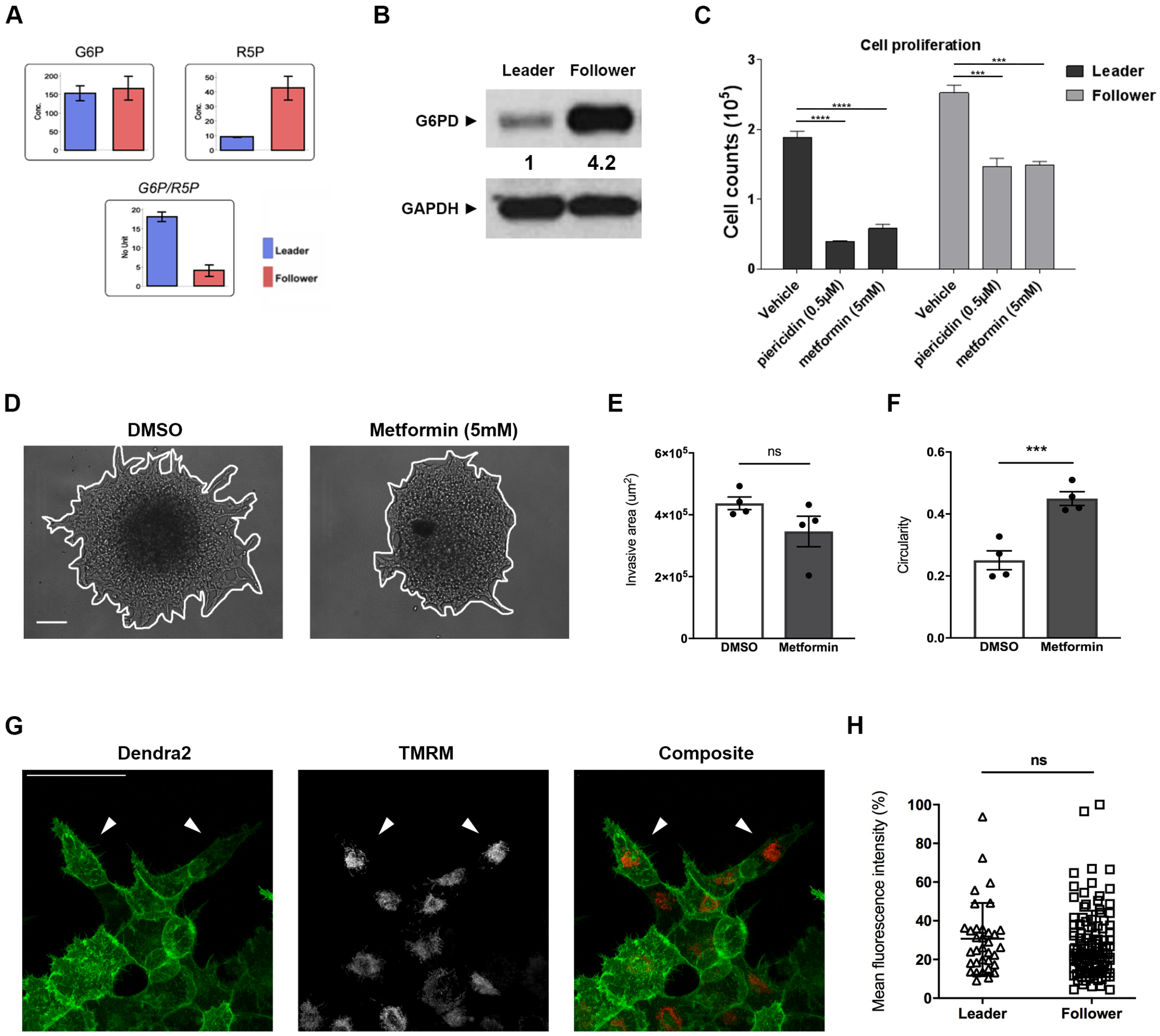
A
G6P
R5P
Conc.
G6P/R5P
No Unit
Leader
Follower
B
Leader
Follower
G6PD ▶
1
4.2
GAPDH ▶
C
Cell proliferation
Cell counts (10^5)
Leader
Follower
****
****
***
***
Vehicle
piericidin (0.5μM)
metformin (5mM)
Vehicle
piericidin (0.5μM)
metformin (5mM)
D
DMSO
Metformin (5mM)
E
Invasive area (um^2)
ns
DMSO
Metformin
F
Circularity
***
DMSO
Metformin
G
Dendra2
TMRM
Composite
H
Mean fluorescence intensity (%)
ns
Leader
Follower

Supplementary Figure 6. PPP is more active in follower cells, and leader cells are more sensitive to mitochondrial complex I inhibitors. (A) Metabolic profiling of leader cells and follower cells was performed and the concentration of metabolites G6P, R5P, and the G6P to R5P ratio were graphed. Error bars represent the mean ± SEM (n=3 biologically independent samples). (B) Cellular lysates prepared from leader cells and follower cells were evaluated for G6PD protein expression with GAPDH as a loading control. Relative densitometry is normalized to leader cells. Repeated 3 times independently with similar results. (C) Leader cells and follower cells were treated with either DMSO, piericidin(0.5μM) or metformin (5mM) for 72 hours then stained with trypan blue and counted by an automated cell counter. (D) H1299 cell spheroids were embedded in Matrigel with either DMSO or 5mM Metformin and allowed to invade for 24 hours. Brightfield representative images are shown. Solid lines designate outer perimeter. Scale bar = 50μm. (E) Invasive area for spheroids shown in D were quantified. Error bars represent the mean ± SEM (n=4 biologically independent samples). (F) Circularity was quantified from the invasive outer perimeter. Error bars represent the mean ± SEM (n=4 biologically independent samples. A two-tailed unpaired Student's t-test was used to analyze statistical significance; p=0.0005. (G) H1299 spheroids expressing Dendra2 were stained with TMRM. Representative confocal images are shown. White arrowheads designate leader cells. Scale bar = 50μm. (H) Quantification of TMRM normalized mean fluorescence intensity. Error bars represent the mean ± SEM (n=30 cells over 3 independent experiments).

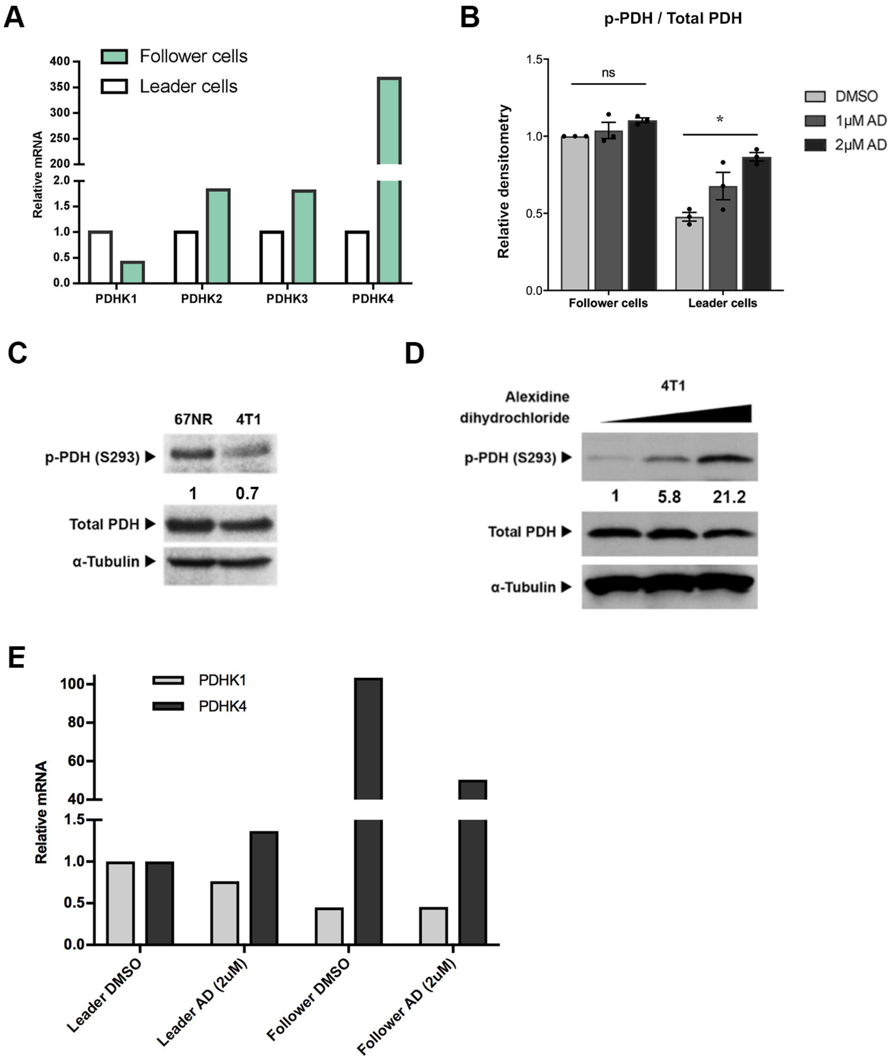

Supplementary Figure 7. Alexidine dihydrochloride induces S293 phosphorylation of PDH. (A) Relative mRNA expression of PDHK1, PDHK2, PDHK3, and PDHK4 in follower cells normalized to leader cell expression levels is shown. Data shown represents the mean (n≥3 biologically independent samples). (B) Relative densitometry normalized to DMSO treated follower cells was averaged from independent experiments (n=3). An ordinary one-way ANOVA with a Tukey's multiple comparisons test was used to determine significance; $p=0.0200$. (C) Cellular lysates of non-metastatic 67NR cells and highly metastatic 4T1 cells were evaluated for p-PDH^{S293}, total PDH, and α-Tubulin protein expression. Relative densitometry normalized to 67NR is shown. Repeated 3 times independently with similar results. (D) 4T1 cells were treated for 24 hours and total protein lysates were evaluated for p-PDH^{S293}, total PDH, and α-Tubulin protein expression. Relative densitometry normalized to DMSO treated control is shown. Repeated 3 times independently with similar results. (E) Relative mRNA expression of PDHK1 and PDHK4 in leader cells and follower cells treated with either DMSO or 2uM alexidine dihydrochloride for 24 hours and normalized to DMSO treated leader cells. Data shown represents the mean (n≥3 biologically independent samples).

	Alexidine (μM)	Bay-876 (μM)	Fraction affected (Fa)	Combination index (CI)
H1299	0.625	0.625	0.45	0.37342
	1.25	1.25	0.14	0.26114
	2.5	2.5	0.1	0.41147
	5	5	0.1	0.82294
	10	10	0.1	1.64588
	20	20	0.1	3.29177
4T1	0.625	0.625	0.26	0.21341
	1.25	1.25	0.08	0.17683
	2.5	2.5	0.08	0.35367
	5	5	0.08	0.70734
	10	10	0.08	1.41468
	20	20	0.06	2.34155
H1792	0.625	0.625	0.16	0.14417
	1.25	1.25	0.15	0.27476
	2.5	2.5	0.15	0.54951
	5	5	0.15	1.09902
	10	10	0.15	2.19804
	20	20	0.1	3.29177
HBEC	0.625	0.625	0.87	0.01574
	1.25	1.25	0.71	1.21772
	2.5	2.5	0.38	618.046
	5	5	0.23	22378.2
	10	10	0.22	56396.8
	20	20	0.19	237131
MRC5	0.625	0.625	0.88	0.01186
	1.25	1.25	0.37	366.961
	2.5	2.5	0.15	93351.1
	5	5	0.13	365085
	10	10	0.09	3854030
	20	20	0.09	7708060
Leader	0.625	0.625	0.95	3.77431
	1.25	1.25	0.41	0.66914
	2.5	2.5	0.15	0.54951
	5	5	0.1	0.82294
	10	10	0.09	1.53148
	20	20	0.09	3.06295
Follower	1.25	1.25	0.43	133.992
	2.5	2.5	0.29	3170.14
	5	5	0.29	6340.27
	10	10	0.27	18915.7
	20	20	0.27	37831.4

Supplementary Table 1. Combination indexes for alexidine dihydrochloride and Bay-876. Combination indexes were performed for parental lung and breast cancer cell lines (H1299, H1792, 4T1), lung subpopulation cell lines (follower and leader cells), and normal lung epithelial cells and lung fibroblasts (HBEC, MRC5) using a 72-hour SRB assay for cell viability as described in the methods. The combinations with synergistic CI values (below 1.0) are highlighted.

3F

GLUT1

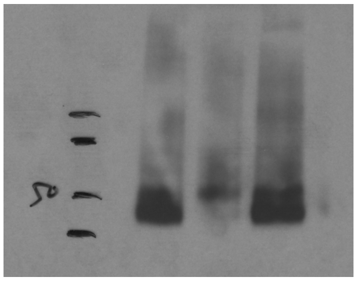

GAPDH

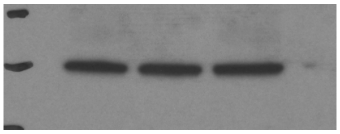

4A

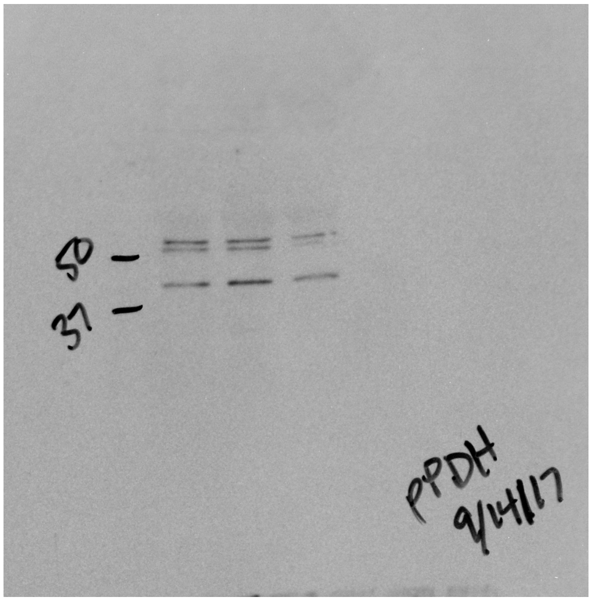

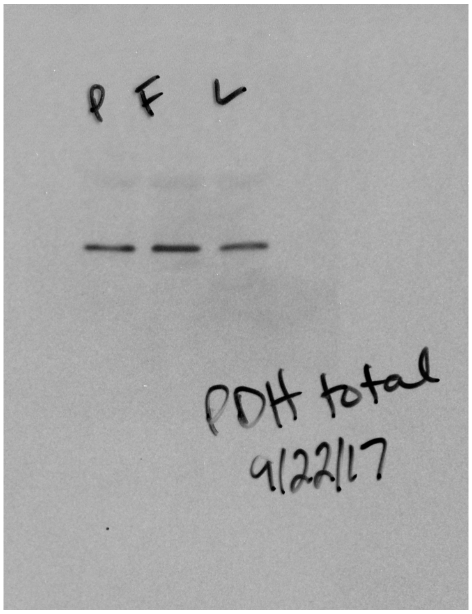

Tubulin

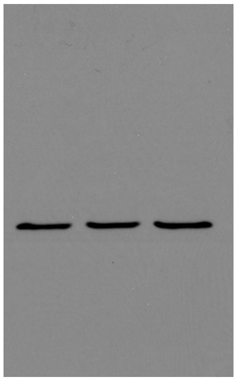

4B

pPDH(S293)

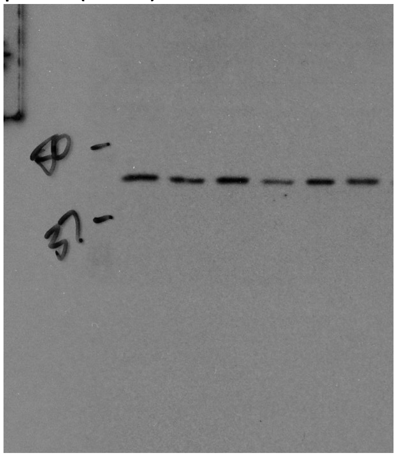

PDH

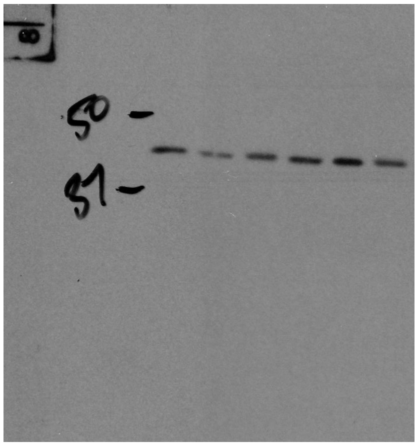

Tubulin

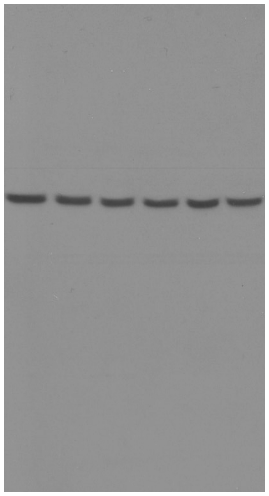

5A

pPDH(S293)

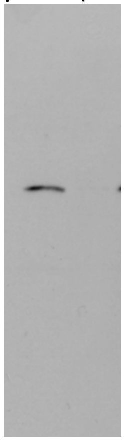

PDH

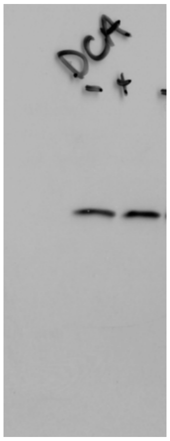

Tubulin

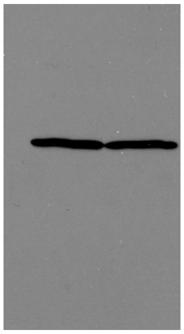

5J

pPDH(S293)

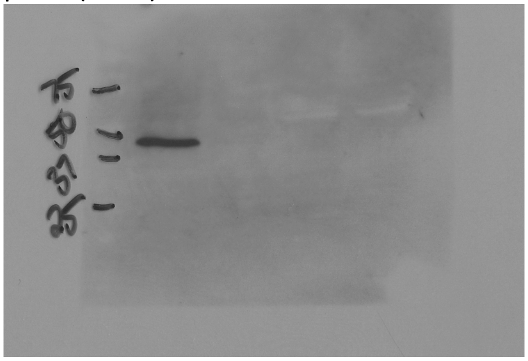

PDH

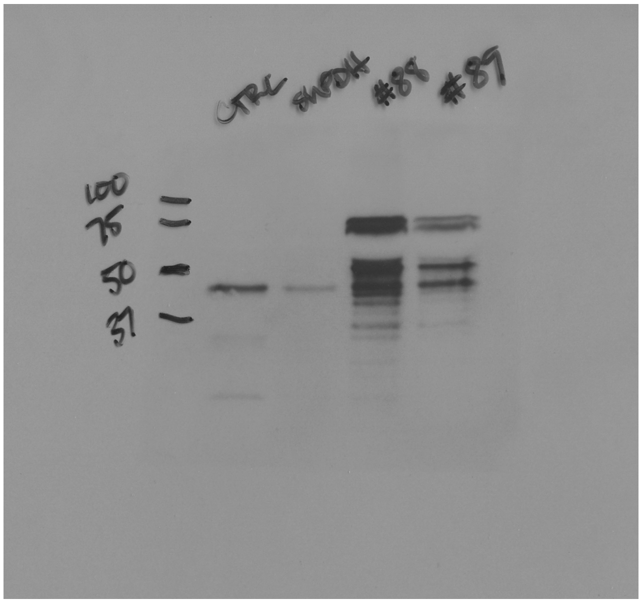

7A

GLUT1

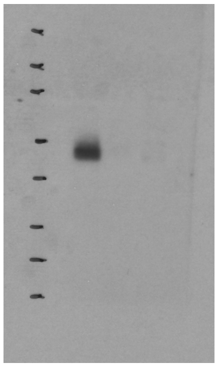

GAPDH

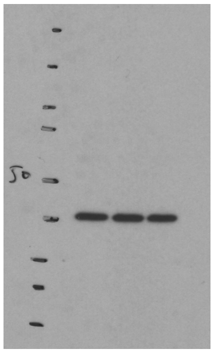

pPDH(S293)

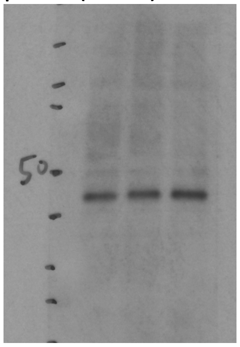

PDH

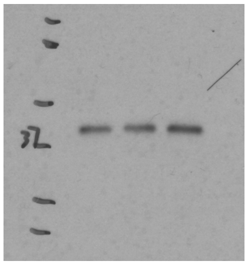

7G

GLUT1

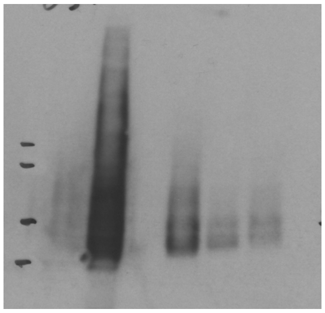

GAPDH

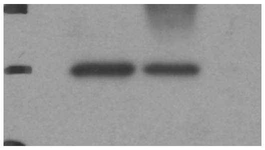

Supplement: Supplementary file 1 — Supplementary Information [file 41467_2020_15219_MOESM1_ESM.pdf]
