# Supplementary material for: Subpopulation targeting of pyruvate dehydrogenase and GLUT1 decouples metabolic heterogeneity during collective cancer cell invasion
Source: Nat Commun. 2020 Mar 24;11:1533. doi: 10.1038/s41467-020-15219-7 (PMC7093428; doi:10.1038/s41467-020-15219-7)
Supplement: Supplementary file 2 — Reporting Summary [file 41467_2020_15219_MOESM2_ESM.pdf]

nature research

Corresponding author(s): Adam I Marcus

Last updated by author(s): Jan 30, 2020

Reporting Summary

Nature Research wishes to improve the reproducibility of the work that we publish. This form provides structure for consistency and transparency in reporting. For further information on Nature Research policies, see Authors & Referees and the Editorial Policy Checklist .

Statistics

For all statistical analyses, confirm that the following items are present in the figure legend, table legend, main text, or Methods section.

n/a	Confirmed	
☐	☒	The exact sample size (*n*) for each experimental group/condition, given as a discrete number and unit of measurement
☐	☒	A statement on whether measurements were taken from distinct samples or whether the same sample was measured repeatedly
☐	☒	The statistical test(s) used AND whether they are one- or two-sided<br>*Only common tests should be described solely by name; describe more complex techniques in the Methods section.*
☒	☐	A description of all covariates tested
☐	☒	A description of any assumptions or corrections, such as tests of normality and adjustment for multiple comparisons
☐	☒	A full description of the statistical parameters including central tendency (e.g. means) or other basic estimates (e.g. regression coefficient) AND variation (e.g. standard deviation) or associated estimates of uncertainty (e.g. confidence intervals)
☐	☒	For null hypothesis testing, the test statistic (e.g. *F*, *t*, *r*) with confidence intervals, effect sizes, degrees of freedom and *P* value noted<br>*Give P values as exact values whenever suitable.*
☒	☐	For Bayesian analysis, information on the choice of priors and Markov chain Monte Carlo settings
☒	☐	For hierarchical and complex designs, identification of the appropriate level for tests and full reporting of outcomes
☒	☐	Estimates of effect sizes (e.g. Cohen's *d*, Pearson's *r*), indicating how they were calculated

Our web collection on statistics for biologists contains articles on many of the points above.

Software and code

Policy information about availability of computer code

Data collection	FACSDiva software (Version 7.0)
Data analysis	ImageJ/Fiji (Version 2.0.0), CellProfiler (Version 3.0.0), CompuSyn (Version 3.0.1), FlowJo (Version 10.2)

For manuscripts utilizing custom algorithms or software that are central to the research but not yet described in published literature, software must be made available to editors/reviewers. We strongly encourage code deposition in a community repository (e.g. GitHub). See the Nature Research guidelines for submitting code & software for further information.

Data

Policy information about availability of data

All manuscripts must include a data availability statement. This statement should provide the following information, where applicable:

- Accession codes, unique identifiers, or web links for publicly available datasets
- A list of figures that have associated raw data
- A description of any restrictions on data availability

The data that support the findings of this study are available from the corresponding authors upon request.

Field-specific reporting

Please select the one below that is the best fit for your research. If you are not sure, read the appropriate sections before making your selection.

☒ Life sciences ☐ Behavioural & social sciences ☐ Ecological, evolutionary & environmental sciences

For a reference copy of the document with all sections, see nature.com/documents/nr-reporting-summary-flat.pdf

Life sciences study design

All studies must disclose on these points even when the disclosure is negative.

Sample size	No sample size calculations were performed. Sample sizes were chosen in order to have at least 3 biologically independent samples per experiment.
Data exclusions	No data were excluded from analyses.
Replication	Experiments were repeated successfully at least 3 times.
Randomization	Either cells or cell spheroids were allocated randomly into experimental groups for drug treatment.
Blinding	Investigators were not blinded to the treatment groups during data collection or analysis because the sample names contained treatment information.

Reporting for specific materials, systems and methods

We require information from authors about some types of materials, experimental systems and methods used in many studies. Here, indicate whether each material, system or method listed is relevant to your study. If you are not sure if a list item applies to your research, read the appropriate section before selecting a response.

Materials & experimental systems

n/a	Involved in the study	
☐	☒	Antibodies
☐	☒	Eukaryotic cell lines
☒	☐	Palaeontology
☒	☐	Animals and other organisms
☒	☐	Human research participants
☒	☐	Clinical data

Methods

n/a	Involved in the study	
☒	☐	ChIP-seq
☐	☒	Flow cytometry
☒	☐	MRI-based neuroimaging

Antibodies

Antibodies used

PDHA1 (9H9AF5) Thermofisher (#459400), dilution 1:1000, Mouse
PDHE1a (pSer293) Millipore (#AP1062), dilution 1:1000 (WB) 1:500 (IF), Rabbit
a-Tubulin (YL1/2) Millipore (#MAB1864), dilution 1:20,000, Rat
GLUT1 Abcam (#ab15309), dilution 1:1000, Rabbit
G6PD Abcam (#ab91034), dilution 1:1000, Mouse
GAPDH (6C5) GeneTex (#GTX41027), dilution 1:30,000 Rat
FAK BD (#610087), dilution 1:1000, Mouse
FAK (pTry397) Thermofisher (#44-642G), dilution 1:1000, Rabbit
MLC (pSer219) Cell signaling (#3671), dilution 1:1000, Rabbit
Mouse secondary Jackson ImmunoResearch (#AB_2338503), dilution 1:10,000
Rabbit secondary Jackson ImmunoResearch (#AB_2313567), dilution 1:10,000
Rat secondary Jackson ImmunoResearch (#AB_2338128), dilution 1:10,000

Validation

Validation of each antibody is provided on the manufacturer's website.

Eukaryotic cell lines

Policy information about cell lines

Cell line source(s)	H1299 and H1792 human NSCLC cells (ATCC), 4T1 (ATCC), and SUM159 human TNBC cells (ATCC). The 67NR cells were obtained from another laboratory.
Authentication	4T1, H1299, H1792, and SUM159 cells were authenticated by STR profiling using our Integrated Genomics Core or by ATCC. The 67NR cell line was not authenticated by STR.
Mycoplasma contamination	All cell lines tested negative for mycoplasma contamination.
Commonly misidentified lines (See ICLAC register)	No commonly misidentified lines were used in this study.

Flow Cytometry

Plots

Confirm that:

- [x] The axis labels state the marker and fluorochrome used (e.g. CD4-FITC).
- [x] The axis scales are clearly visible. Include numbers along axes only for bottom left plot of group (a 'group' is an analysis of identical markers).
- [x] All plots are contour plots with outliers or pseudocolor plots.
- [x] A numerical value for number of cells or percentage (with statistics) is provided.

Methodology

Sample preparation	Tissue culture cells were harvested in EDTA-free trypsin and then stained.
Instrument	BD FACSCanto-II cytometer
Software	FACSDiva software (Version 7.0), FlowJo (Version 10.2)
Cell population abundance	Between 10,000 cells and 100,000 cells were acquired per sample, and the total population was analyzed.
Gating strategy	All cells were analyzed using SSC/Pacific blue separation, with the gate drawn around the Pacific blue positive population.

- [x] Tick this box to confirm that a figure exemplifying the gating strategy is provided in the Supplementary Information.
